# Effects of Nutritional Supplements on Endurance Performance and Subjective Perception in Athletes Exercising in the Heat: A Systematic Review and Network Meta-Analysis

**DOI:** 10.3390/nu17132141

**Published:** 2025-06-27

**Authors:** Jiahao Li, Shuning Liu, Siqi Wang, Yutong Wu, Liu Yang, Qi Luo, Zixiao Li, Shengxin Yang, Kai Zhao, Chang Liu

**Affiliations:** 1 School of Leisure Sports and Tourism, Beijing Sport University, Beijing 100084, China; 2022012043@bsu.edu.cn (J.L.); wangsiqi@bsu.edu.cn (S.W.); 2 College of Sports Science, Beijing Sport University, Beijing 100084, China; 2023013553@bsu.edu.cn (S.L.); 2024012786@bsu.edu.cn (Y.W.); 2023011843@bsu.edu.cn (L.Y.); 2024012816@bsu.edu.cn (S.Y.); 3 China Football Academy, Beijing Sport University, Beijing 100084, China; 2023010631@bsu.edu.cn; 4 School of Sports Medicine and Rehabilitation, Beijing Sport University, Beijing 100084, China; cynthiaxiaozili@bsu.edu.cn; 5 China Volleyball Academy, Beijing Sport University, Beijing 100084, China

**Keywords:** endurance performance, thermal comfort, athletic performance, thermoregulation, nutritional supplements, network meta-analysis

## Abstract

**Objectives**: This study aimed to evaluate the efficacy of various nutritional supplements in enhancing endurance performance and subjective thermal perception in athletes exposed to high-temperature environments through a systematic review and network meta-analysis. **Methods**: A comprehensive search was conducted in PubMed, Embase, Web of Science, Cochrane Library, and EBSCOhost from inception to January 2025. Studies were included if they evaluated the effects of nutritional supplements on either endurance performance or subjective thermal perception in athletes under heat stress. Two independent reviewers screened the literature, extracted data, and assessed the risk of bias. A network meta-analysis was performed using R software (version 4.3.1). The search was limited to English-language publications and employed both MeSH and free-text terms related to “athletes,” “nutritional supplements,” and “exercise performance,” using Boolean operators (AND/OR) to construct the strategy. **Results**: Twenty-five randomized controlled trials (RCTs) involving 552 participants were included, yielding 22 comparisons: 18 assessed endurance performance, and 11 assessed subjective perception. Standardized mean differences (SMDs) and posterior probabilities (P-scores based on Bayesian ranking) were calculated using random-effects and Bayesian models. Menthol (SMD = −1.83, 95% CI [−3.15, −0.51]; P-score = 71.04%) and taurine (SMD = 0.91, 95% CI [0.08, 1.73]; P-score = 12.75%) demonstrated significant positive effects on endurance. Menthol energy gel showed the greatest improvement in thermal comfort (SMD = 2.14, 95% CI [1.01, 3.26]; P-score = 99.54%). **Conclusions**: Menthol and taurine appear effective in enhancing endurance in hot environments, while menthol energy gel substantially improves perceived thermal comfort. Future research should apply stricter controls regarding environmental conditions, supplement dosage, and participant characteristics. While individual supplements may offer limited benefits, synergistic combinations may yield greater improvements in performance and comfort.

## 1. Introduction

With global warming and the increasing frequency of international large-scale competitions held in hot environments, the issue of athletic exercise performance in heat stress has received growing attention. High-temperature environments (ambient temperature ≥ 27 °C) pose significant challenges to athletes’ physiological functions, especially in terms of endurance performance and subjective perception [1]. According to controlled laboratory studies examining time-to-exhaustion and power output during endurance cycling in high-temperature environments, male athletes experienced an average performance decline of 17.7% and females 12.4%, primarily due to impaired thermoregulation and increased cardiovascular strain [2,3].

Heat impairs exercise performance by affecting central fatigue regulation, leading to reduced time to exhaustion and a decline in maximal voluntary contraction (MVC) [2]. Compared to 17 °C, 22 °C, and 27 °C, the average power output of athletes is significantly reduced at 37 °C, along with a shortened time to volitional exhaustion [2]. High temperature and humidity markedly increase the body’s thermal load, causing elevated core temperature, accelerated heart rate, and decreased cardiac output [3] while simultaneously intensifying subjective fatigue and thermal stress [4].

For instance, when athletes cycled at a matched workload for 60 min in 35 °C versus 20 °C, mean RPE scores were 2–3 points higher on the 6–20 Borg scale, and excessive thermal stress severely challenges both physiological function and subjective perception [5] (When exercising at the same intensity and duration in 35 °C heat versus 20 °C ambient conditions, athletes’ RPE increased significantly by ≥2 points within the first 10 min and remained 2–3 points higher than the temperate group throughout the 60 min session). In high heat and humidity, time to exhaustion is significantly reduced and blood oxygen saturation declines [6]. These findings highlight the negative effects of heat on endurance performance and perceived exertion in athletes.

To alleviate heat stress and subjective fatigue and optimize exercise performance, several strategies have been proposed, including (i) cooling interventions [7], (ii) vibration stimulation therapy [8], and (iii) nutritional supplementation [9]. In terms of nutritional strategies, various supplements such as creatine [10], branched-chain amino acids (BCAA), carbohydrates [11], caffeine [12], and beetroot juice [13] have been shown to exert potential ergogenic effects in temperate or cold environments. Although multiple nutritional interventions have been proposed, most existing RCTs have been conducted under thermoneutral conditions.

There is a lack of systematic investigation into the efficacy of nutritional supplementation in alleviating perceptual fatigue and enhancing exercise performance in heat stress, and the effects of such interventions vary considerably. Some studies demonstrate that caffeine [14] and nitrates [15] can impair endurance performance and thermoregulatory perception under heat stress. Given this evidence of potential detrimental effects in the specific environmental conditions relevant to our study, we excluded RCTs of these single supplements from our primary analysis.

In contrast, other supplements such as BCAA [16] and taurine [17] may help delay or improve central fatigue and thermal perception induced by heat. However, the effects of many nutritional supplements under heat stress remain unclear. Some studies have reported performance decrements with caffeine or nitrate during exercise in the heat; however, single-ingredient trials of these agents were excluded for methodological reasons explained in Section 2.3, not because of any presumed benefit or harm.

To address the above issues and fill this gap, we employed a network meta-analysis (NMA) to compare the direct and indirect effects of various interventions. Unlike the study by Peel et al. (2021), which analyzed only 9 supplements and focused primarily on core temperature as the physiological outcome, our study includes 16 interventions and, for the first time, integrates subjective thermal discomfort indices into the network model, offering a novel perspective for comparing nutritional supplements [18].

Given the diversity of supplements and the limited direct comparisons, a comprehensive evidence synthesis was needed to inform decision-making [19]. To compare the effects of different nutritional supplements on endurance performance and subjective perception under heat stress, this study used a network meta-analysis approach to evaluate the direct and indirect effects of multiple interventions and rank their effectiveness. This provides valuable evidence to help athletes and coaches select optimal nutritional strategies for performance enhancement in hot environments.

## 2. Materials and Methods

### 2.1. Protocol and Registration

This systematic review and network meta-analysis was designed and reported in accordance with the Preferred Reporting Items for Systematic Reviews and Meta-Analyses [20] (PRISMA 2020), the PRISMA extension statement for network meta-analyses, the Cochrane Handbook for Systematic Reviews of Interventions, and guidelines from the National Institute for Health and Care Excellence [21]. The protocol was prospectively registered in the International Prospective Register of Systematic Reviews (PROSPERO) in May 2025 (registration number CRD420251051588).

### 2.2. Search Strategy and Information Sources

This network meta-analysis followed the PRISMA-NMA guidelines. A systematic search was performed in five biomedical databases from inception to 1 May 2025, including PubMed, Embase, Web of Science, Cochrane Library, and EBSCOhost. The search was restricted to English-language publications. The search strategy combined MeSH terms and free-text terms, focusing on the three key themes of athletes, nutritional supplements, and exercise performance. Boolean operators (OR/AND) were used to construct the search logic. Full search strategies are available in Supplementary Figure S1. The search was developed collaboratively by multiple reviewers (J.L., S.L., L.Y., Y.W., S.Y., Z.L., Q.L.) and optimized using the Polyglot Search Translator from the SR-Accelerator platform (https://sr-accelerator.com/, accessed on 15 May 2025). Citation tracking and manual searching of relevant primary studies and reviews were also conducted. After removing duplicates, eligible studies were identified via a three-stage screening process (title, abstract, and full text) conducted manually.

### 2.3. Inclusion and Exclusion Criteria

Studies that assessed the effects of oral nutritional supplements on endurance performance or subjective perception (e.g., RPE, thermal comfort) under high-temperature conditions (≥27 °C) were eligible for inclusion. The inclusion criteria followed the PICOS framework:

Inclusion criteria: Population (P): Athletes (elite or non-elite) engaged in endurance or high-intensity team sports, regardless of age or sex, under high-temperature conditions (ambient temperature ≥ 27 °C). Only trials that employed acute or short-term (≤14 days) oral supplementation protocols were eligible; studies evaluating chronic supplementation were excluded. Intervention (I): Oral nutritional supplements including but not limited to creatine, BCAA, carbohydrates, carbohydrate + protein combinations, electrolytes, tyrosine, menthol, taurine, caffeine + taurine combinations, ginseng, creatine + glycerol combinations, polyphenol antioxidants, astaxanthin, and carbonated water. Comparison (C): Placebo control, no supplement, head-to-head comparisons between different supplements, or the same supplement with different doses or timings. Outcomes (O): Primary outcome 1: Endurance performance indicators such as time to exhaustion (TTE), time trial performance, exercise distance, sprint performance, and power output. Primary outcome 2: Subjective perception measures including RPE, fatigue, thermal sensation, and recovery perception. Study design (S): Randomized controlled trials (RCTs) or well-designed quasi-RCTs with at least two intervention arms.

Exclusion criteria: (1) Non-human studies; (2) non-randomized or observational designs; (3) studies conducted in thermoneutral or cold environments (ambient < 27 °C); (4) nutritional interventions delivered non-orally (e.g., intravenous); (5) trials lacking extractable outcome data; (6) duplicate publications or conference abstracts without full text; (7) studies rated as “high risk of bias” in ≥1 RoB 2 domain.

The rationale for excluding caffeine and nitrate monotherapies—single-ingredient caffeine and nitrate trials were excluded a priori. Pilot mapping showed that more than 40% of all eligible heat-exercise RCTs compared caffeine or nitrate solely with placebo yet provided almost no head-to-head links with the other 14 supplements considered in this review. Including these trials would fragment the evidence into two disconnected subnetworks, violating the single-network requirement for valid indirect comparisons. In addition, acute caffeine dosing (3–9 mg·kg^−1^, single bout) and chronic nitrate loading (≥3 days) differ mechanistically and temporally from the largely single-dose, non-vasoactive agents analyzed here, thereby breaching the transitivity assumption. Finally, two recent network meta-analyses [22,23] have already synthesized caffeine and nitrate under comparable heat-stress conditions; duplicating those findings lay outside the scope of the present work.

### 2.4. Study Selection

**Figure 1:**
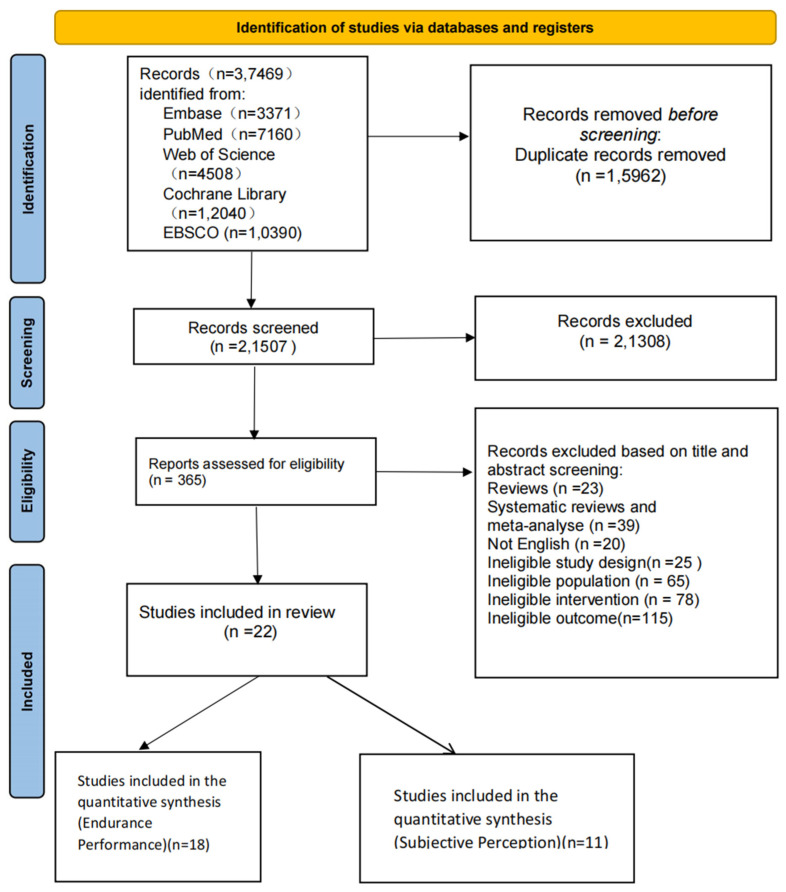
Screening flowchart.

Three reviewers (J.L., S.L., and C.L.) independently screened titles, abstracts, and full texts and determined eligibility based on predefined criteria. Disagreements were resolved by discussion or with input from a fourth reviewer (L.Y.). The study selection process followed the PRISMA flow diagram (Figure 1). All records were managed using EndNote 21 software.

### 2.5. Data Extraction and Processing

Key data were independently extracted by two reviewers (S.L. and J.L.), with discrepancies resolved by a third reviewer (C.L.). Extracted information included author, publication year, country or region, sample size, sex, mean age, type of sport, supplement type and dosage, duration of intervention, heat exposure parameters (temperature/humidity), outcome type (endurance performance or subjective perception), test methods, and main findings.

All quantitative data were extracted directly from tables or text in the original articles. For endurance performance outcomes, we prioritized post-intervention mean ± standard deviation (SD) values. For subjective perception outcomes (e.g., RPE), data were extracted in the same format where available. If complete mean ± SD data were unavailable and could not be retrieved from the full text, the study was retained for narrative synthesis but excluded from the quantitative network meta-analysis [24].

All data were compiled in Microsoft Excel 2021 and standardized (e.g., unit conversions, unified naming conventions) for subsequent statistical modeling and network structure construction.

### 2.6. Bias Risk Assessment Statement

The risk of bias in RCTs was evaluated using the Cochrane Risk of Bias Tool 2.0 (RoB 2). Two reviewers (S.L. and J.L.) independently assessed risk of bias across five domains: randomization process, deviations from intended interventions, missing outcome data, measurement of the outcome, and selective reporting. Discrepancies were resolved through discussion or by consulting a third reviewer (C.L.). The confidence in the evidence was further evaluated using the Confidence in Network Meta-Analysis (CINeMA) approach [24].

### 2.7. Statistical Analysis

Following PRISMA-NMA guidelines [25], all analyses were conducted under the frequentist framework [26] using random-effects models in R 4.3.1. Standardized mean differences (SMD) and their 95% confidence intervals (CI) were used as the effect size metric due to variations in outcome measurement units across studies.

Statistical significance was defined solely by whether the 95% confidence interval (CI) of the SMD excluded zero. P-scores (0–1) were used only to rank treatments on the probability of being best and do not in themselves denote statistical significance.

Network diagrams were constructed to visualize the evidence geometry, with nodes representing interventions and edges denoting direct comparisons. Edge thickness scaled with the number of trials, while node size was proportional to the total sample size. Inconsistency assessment included global loop evaluation through inconsistency factors (IF) with 95% CI [27]. Local node-splitting analysis quantifying disagreement between direct and indirect evidence. The node-splitting method revealed non-significant inconsistency across all comparisons (all *p* > 0.05) [28]. Given the absence of significant inconsistency, a consistency model was adopted for all analyses.

Ranking of interventions was performed using surface under the cumulative ranking curve (SUCRA) values, which range from 0 (worst) to 1 (best) [19,26].

All network meta-analyses and publication bias assessments were performed in R 4.3.1. For Bayesian consistency models, the gemtc package was used with JAGS. Four Markov chains were run with initial values set to 2.5. The adaptation phase (n.adapt) was set to 50,000 iterations, and the sampling phase (n.iter) to 200,000 iterations, with a thinning interval of 10. Model convergence was evaluated through trace and density plots.

Intervention rankings were quantified using P-scores, with graphical outputs (network plots, forest plots, SUCRA) generated in R. Publication bias assessment integrated visual and statistical approaches: funnel plots (standard error vs. SMD) enabled asymmetry detection where symmetry suggests minimal bias, complemented by three quantitative tests: Egger’s regression for small-study effects. Begg–Mazumdar rank correlation for publication selection. Thompson–Sharp test for heterogeneity-related bias. Using α = 0.05 as the significance threshold, non-significant *p*-values, and symmetrical funnel patterns collectively indicated low publication bias risk. Bayesian prior and convergence details are provided in Section 3.4.

Assessment of Transitivity:

We assessed the transitivity assumption by comparing potential effect modifiers across comparisons, including participant demographics, heat exposure parameters, intervention types, and outcome definitions. No substantial clinical or methodological heterogeneity was found that would undermine the validity of indirect comparisons.

## 3. Results

### 3.1. Study Selection and Characteristics

According to the PRISMA 2020 flowchart (Figure 1), a total of 37,469 records were initially retrieved. After removing duplicates, 21,507 records remained. Following title and abstract screening, 365 articles were assessed in full text, and 25 RCTs met the inclusion criteria for the systematic review. Among them, 22 studies were eligible for inclusion in the network meta-analysis.

A total of 552 participants were included across the 25 RCTs, predominantly healthy male athletes aged 18–35 years. The average age was 28.3 ± 4.8 years. Participants engaged in a range of endurance (e.g., running, cycling, rowing) and intermittent sports (e.g., football and rugby), ensuring good representativeness and heterogeneity across training levels.

All studies were conducted in hot environments, with ambient temperatures ranging from 27 °C to 40 °C and relative humidity between 40 and 80%. The interventions were all oral nutritional supplements, mostly administered acutely (single or short-term intake), with a few studies using chronic supplementation (>3 days). Seventeen discrete oral supplementation protocols were identified (study counts in brackets): creatine (5), BCAA (2), tyrosine (3), taurine (1), polyphenol antioxidants (2), carbohydrate–protein blends (1), electrolyte/sodium-based supplements (4), multi-ingredient formulations (3), menthol/ice slurries (3), and cold-water control (1).

Representative formulations were creatine monohydrate and creatine + glycerol; leucine-rich BCAA drinks; L-tyrosine; taurine powder; quercetin/catechin polyphenols; maltodextrin + whey isolate carbohydrate–protein blends; high-sodium (~70 mmol L^−1^), low-sodium (~20 mmol L^−1^) and sodium-citrate loading; caffeine + taurine and caffeine + Panax ginseng multi-ingredient mixes; menthol mouth-rinse, menthol energy gel, and ice + menthol slurry cooling agents. An isocaloric flavored water or cold-water rinse served as the placebo/no-supplement reference in every trial (see Table 1 and Supplement Figure S2 for full dosing schedules)

Endurance performance outcomes (e.g., time to exhaustion, time trials, power output) were reported in all studies, while 11 studies also reported perceptual outcomes (e.g., Borg RPE, thermal sensation, fatigue scores) using standardized international assessment tools, summarized in Table 1.

### 3.2. Risk of Bias Assessment

**Figure 2:**
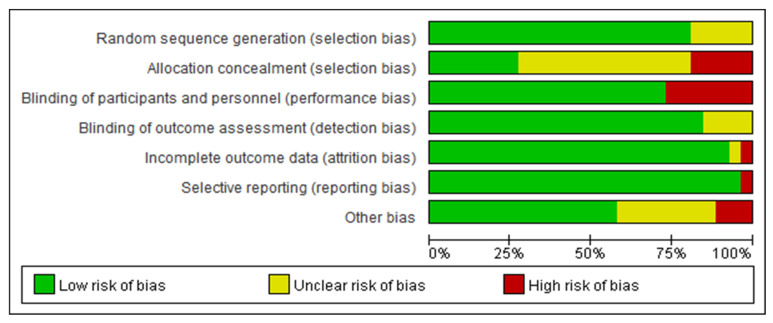
Bias assessment.

All included RCTs were assessed using the Cochrane Risk of Bias Tool (RoB 2.0). Approximately 90% reported appropriate randomization methods; 50% adequately described allocation concealment; and over 75% employed participant and assessor blinding (Figure 2). Outcome assessment was largely objective, and dropout rates were low. Overall, the studies demonstrated moderate to high methodological quality, with few showing serious concerns about bias.

### 3.3. Network Geometry

**Figure 3:**
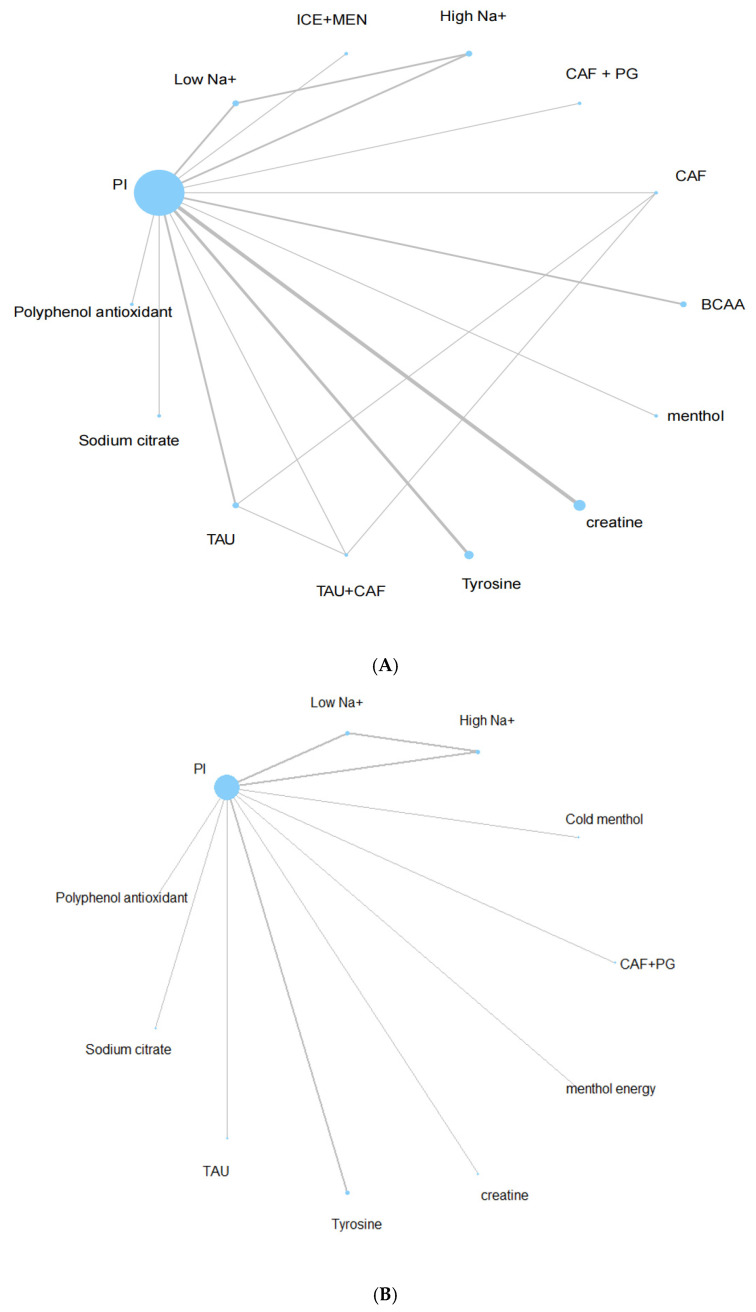
(**A**) Endurance sports performance network plot. (**B**) Subjective perception network plot.

Two separate networks were constructed for the outcomes of endurance performance and subjective perception (Figure 3A,B). Each node represents a unique intervention, and the edges indicate direct comparisons. Node size reflects sample size, while edge thickness indicates the number of trials comparing the linked interventions.

The endurance performance network included 14 interventions and was well connected, allowing both direct and indirect comparisons via placebo intervention (PI) as the reference comparator. The perceptual network was slightly sparser, including 11 interventions, but still sufficiently connected for network analysis.

### 3.4. Model Convergence

Trace and density plots indicated that all Markov chains reached convergence after 50,000 burn-in iterations. Potential scale reduction factors (PSRFs) approached 1.00, confirming satisfactory model fit and convergence. In the Bayesian framework, we used non-informative priors for all parameters: a normal distribution with mean 0 and variance 10,000 was applied to treatment effect estimates, and a uniform distribution U (0, 5) was specified for the between-study standard deviation (τ), consistent with recommendations for Bayesian NMA. Four Markov chains were run with an adaptation phase of 50,000 iterations followed by 200,000 sampling iterations.

The node-splitting analysis revealed no statistically significant inconsistency between direct and indirect comparisons across the network for both endurance performance and subjective perception outcomes (all *p*-values > 0.05), supporting the assumptions of transitivity and coherence.

**Figure 4:**
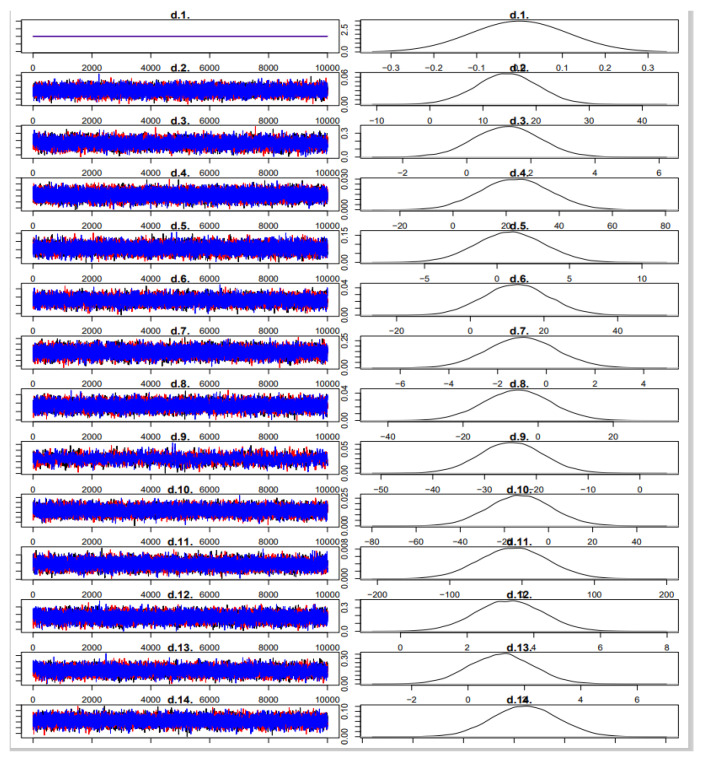
Iterative posterior distribution of endurance sports performance.

**Figure 5:**
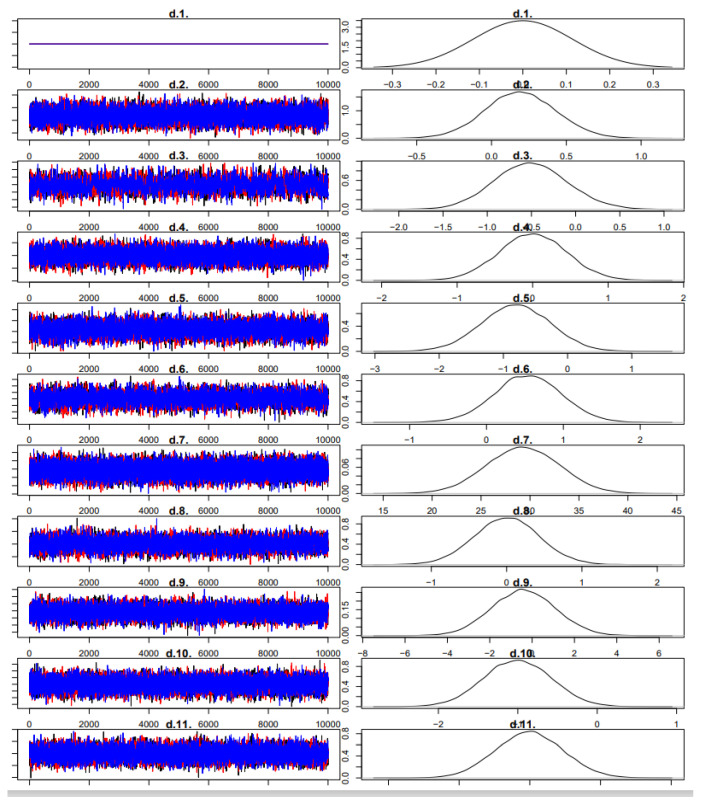
Subjective perception iteration and posterior distribution map.

Convergence was assessed using trace plots and the potential scale reduction factor (PSRF), with values < 1.05 indicating good convergence. All parameters reached satisfactory convergence (PSRF ≈ 1.00). Detailed trace plots and model diagnostics have been provided in the Figure 4 and Figure 5).

No additional iterations were required. See Figure 4 and Figure 5 for details.

### 3.5. Network Meta-Analysis Outcomes

#### 3.5.1. Endurance Performance

Unless otherwise stated, an effect was deemed statistically significant when its 95% CI did not cross zero; P-score values are presented for ranking purposes only. Eighteen studies reported endurance performance involving fourteen interventions. Heterogeneity was moderate (*I*^2^ = 43.2%, 95% CI: 0–72.8%). Consistency checks showed no significant inconsistency (Q total = 15.84, *p* = 0.07; Q design = 14.65, *p* = 0.066; Q inconsistency = 1.20, *p* = 0.274).

**Figure 6:**
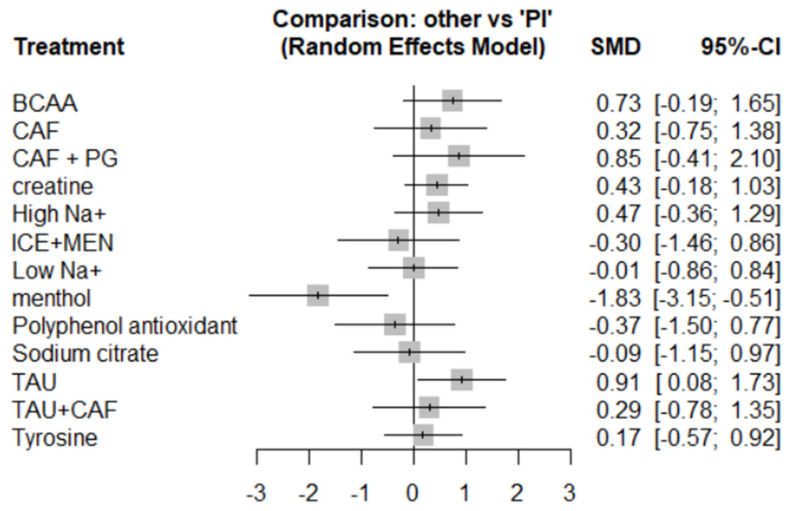
Forest map of endurance sports performance.

**Figure 7:**
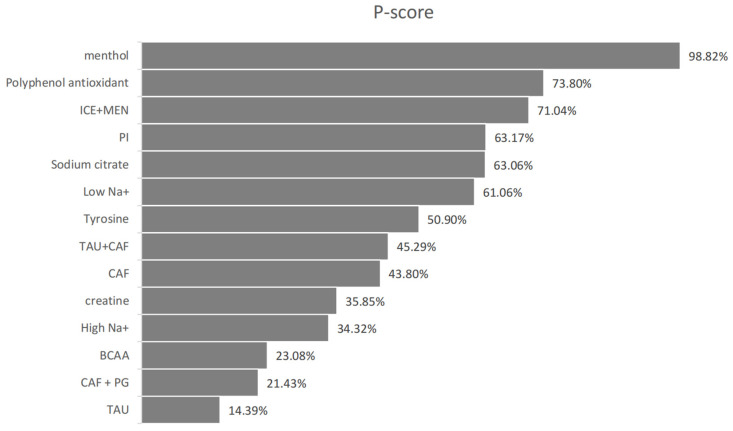
Endurance sports performance P-score.

**Figure 8:**
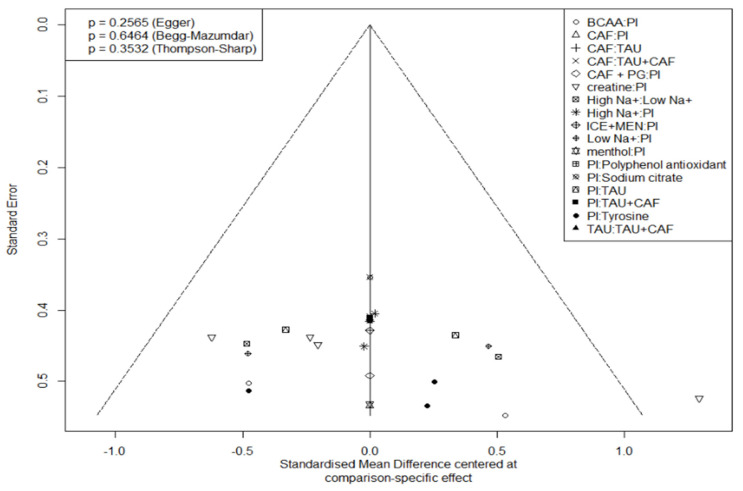
Funnel chart of endurance sports performance research.

The direction of SMDs was defined such that a positive value indicates improvement for outcomes like power output or distance, and a negative value indicates improvement for outcomes where lower values are favorable, such as time-to-exhaustion. Menthol (MEN) showed a significant improvement in endurance performance (SMD = –1.83, 95% CI: –3.15 to –0.51, P-score = 98.82%), where negative SMD reflects shorter completion time (e.g., in 5 km time trials) and, thus, superior performance. Taurine (SMD = 0.91, 95% CI: 0.08 to 1.73, P-score = 14.39%) also showed statistically significant improvement. (Please refer to Figure 6, Figure 7 and Figure 8 for details).

Other supplements like BCAA (SMD = 0.73), creatine (SMD = 0.43), and high-sodium (SMD = 0.47) showed positive trends but lacked statistical significance due to wide confidence intervals. ICE + Menthol and polyphenols also exhibited non-significant effects (SMDs –0.30 and –0.37, respectively). Supplements such as sodium citrate, low-sodium, tyrosine, and caffeine-based combinations (CAF, CAF + PG, TAU + CAF) showed minimal or uncertain effects (SMDs close to zero; wide CIs).

#### 3.5.2. Subjective Perception

Unless otherwise stated, an effect was deemed statistically significant when its 95% CI did not cross zero; P-score values are presented for ranking purposes only. Eleven studies assessed perceptual outcomes across 11 interventions. Moderate heterogeneity (*I*^2^ = 50.4%) with non-significant between-study inconsistency (Cochran’s Q = 6.05, *p* = 0.109) supported the application of a random-effects model.

**Figure 9:**
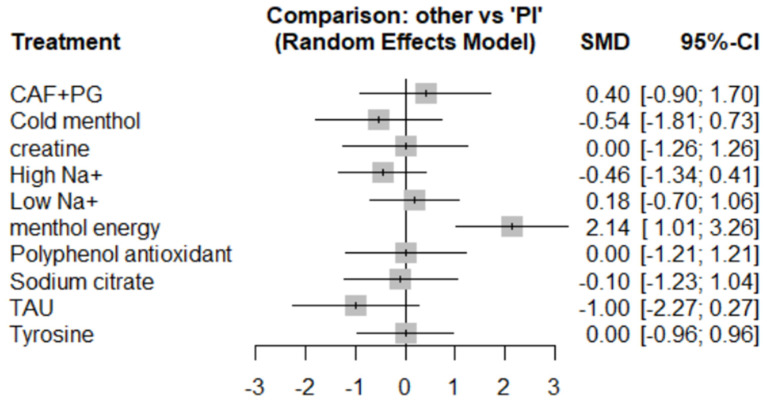
Subjective perceived forest map.

**Figure 10:**
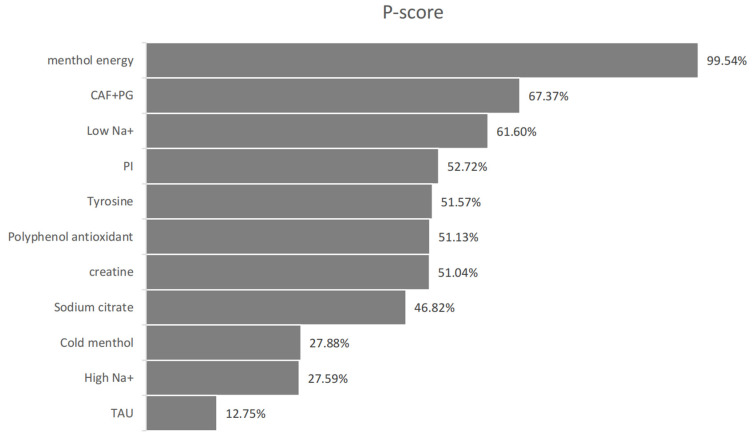
Subjective perceived P-score.

**Figure 11:**
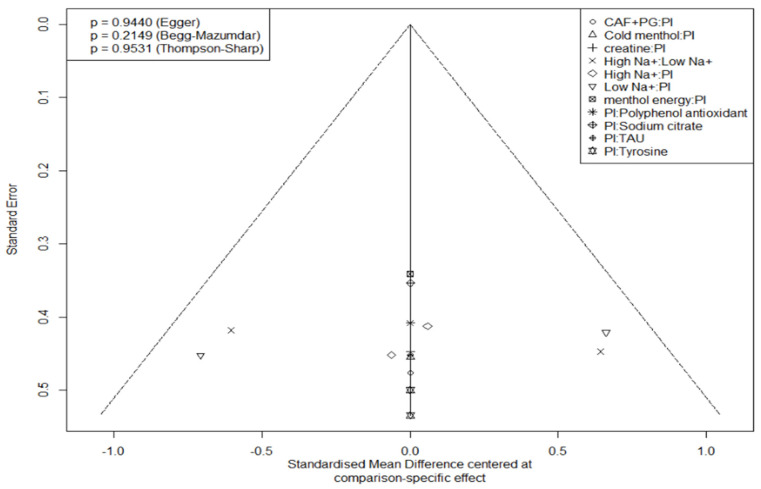
Subjective perception funnel diagram.

Menthol energy gel showed a significant positive effect (SMD = 2.14, 95% CI: 1.01 to 3.26, P-score = 99.54%). CAF + PG (SMD = 0.40, 95% CI: –0.90 to 1.70) and low sodium (SMD = 0.18, 95% CI: –0.70 to 1.06) showed weak positive trends. High sodium (SMD = –0.46) and cold menthol (SMD = –0.54) demonstrated no significant effects. (Please refer to Figure 9, Figure 10 and Figure 11 for details).

Taurine unexpectedly showed a negative trend (SMD = –1.00, 95% CI: –2.27 to 0.27), potentially due to dose variations or limited sample sizes. Creatine, tyrosine, and polyphenols all showed null effects (SMD = 0.00), suggesting their mechanisms may be less effective in thermally stressful conditions.

### 3.6. Sensitivity Analysis

Removing small-sample studies (n < 10) or switching random-effect models did not significantly alter the key findings. Menthol (SMD = –1.83), taurine (SMD = 0.91), and menthol energy (SMD = 2.14) retained their statistical significance with consistent effect sizes and confidence intervals.

Publication bias was not evident in either domain (Egger’s test: endurance *p* = 0.2565, perception *p* = 0.9440). Thompson–Sharp heterogeneity test also indicated acceptable between-study variance.

Score rankings were consistent with the effect size direction, though some high-ranking supplements had non-significant SMD, indicating the need for cautious interpretation.

Publication bias was “not evident” in either domain. “Funnel plots appeared largely symmetrical, with no signs of small-study effects”, and Egger’s tests were non-significant (endurance *p* = 0.2565; perception *p* = 0.9440). Thompson–Sharp tests likewise indicated acceptable heterogeneity, reinforcing a low risk of publication bias.

## 4. Discussion

This study is the first NMA to systematically evaluate the effects of different nutritional supplements on endurance performance and subjective perception in athletes under high-temperature conditions. As of 1 May 2025, no comprehensive summary of nutritional interventions specifically targeting endurance and perceptual responses in hot environments had been published. Our main findings suggest the following:

(1) Menthol and taurine show positive effects in improving subjective thermal comfort and endurance performance;
(2) Among all interventions, menthol energy gels demonstrated the most robust potential for enhancing subjective perception under heat stress;
(3) In high-temperature environments, the combined use of supplements appeared to be more effective than single-agent interventions.

In contrast, supplements such as sodium citrate, high sodium, and low sodium showed limited or even potentially negative effects (e.g., SMD = –0.01, 95% CI [–0.86, 0.84]). While some previous studies have explored the effects of individual supplements in hot conditions, our network-based approach allowed for simultaneous comparison of multiple agents and endpoints, suggesting that combining multiple supplements may be more effective than using a single supplement alone. This provides a foundation for future research to optimize supplement regimens for endurance performance in the heat. Consequently, the present network meta-analysis offers probabilistic comparisons rather than prescriptive rankings; the findings are hypothesis-generating and should guide targeted future research under more homogeneous conditions.

### 4.1. Overview of Mechanisms and Research Status of Nutritional Supplements in Heat

BCAA theoretically alleviates central fatigue by suppressing central serotonin synthesis, and some studies have observed a reduction in RPE scores under heat stress. However, direct improvements in endurance performance remain inconsistent, potentially due to variations in dosing and timing [46]. TAU, a conditionally essential amino acid with antioxidant, membrane-stabilizing, and calcium-regulating functions, has attracted increasing attention in heat-stress contexts. This study found taurine to significantly enhance endurance performance in hot environments, possibly by mitigating heat-induced central fatigue and emotional disturbances. However, its effect on subjective perception was not statistically significant, which may be due to the limited number of relevant studies [49,54].

Tyrosine, a precursor to dopamine and norepinephrine, may enhance central nervous system regulation under heat stress. However, results were neutral in this review, likely due to small sample sizes and inconsistent intervention protocols [42,43].

Antioxidants such as polyphenols and astaxanthin may combat heat-induced oxidative stress, maintain mitochondrial function, and delay fatigue onset. Although our meta-analysis did not find statistically significant effects, their application in heat adaptation training and long-term hot exposure scenarios remains promising [50].

Creatine primarily acts on the phosphagen system and may improve cellular hydration and thermal stability [48,55]. However, it remains controversial in heat, as weight gain and impaired heat dissipation may negate its benefits. Thus, individualized assessment is needed for use under heat stress [29,30,32,48].

Caffeine, although well-established as a performance enhancer under temperate conditions, may impair performance in the heat due to elevated heart rate and core temperature. In this study, most caffeine supplements were combined with taurine or ginseng, showing potential synergistic effects on subjective perception, but further validation is needed [43,56,57].

Electrolyte and alkaline supplements (e.g., sodium bicarbonate, high-sodium drinks) may help maintain fluid balance and buffer capacity. Some studies suggest benefits in prolonging endurance under heat, but no significant effects were observed in our analysis [39,41].

Menthol-based supplements activate TRPM8 cold receptors and improve thermal sensation and RPE scores. They are well-accepted in hot and humid environments for enhancing subjective comfort. However, effects on objective performance vary across studies, possibly due to differences in concentration, administration method (e.g., mouth rinse, ingestion, topical), and exercise intensity. Some evidence also suggests that the cooling sensation of menthol may mask physiological strain, potentially increasing central fatigue [36,38].

Overall, taurine, antioxidants, and menthol supplements show promising effects on both subjective perception and endurance in hot conditions. In contrast, BCAA, creatine, and sodium-based supplements show variable results, and caffeine requires careful use. Future research should emphasize precise supplement design based on environmental conditions, individual heat tolerance, and sport-specific demands.

### 4.2. Effects on Endurance Performance

This study used a random-effects model to analyze the impact of various nutritional supplements on exercise performance under high-temperature conditions. The results revealed significant differences in effectiveness among the supplements. Menthol showed the most pronounced negative standardized mean difference (SMD) value. This is because, in the 5 km time trial, the outcome metric was defined such that “shorter time indicates better performance.” Therefore, when the menthol group (intervention group) had a significantly shorter completion time than the control group, the mean difference (intervention mean—control mean) was negative, resulting in a negative SMD. This outcome aligns with the directional definition of the metric [49], suggesting that menthol may effectively improve performance in hot environments by enhancing thermal comfort or delaying perceived fatigue [58,59,60].

Taurine (TAU) exhibited a statistically significant positive effect. According to current research, its beneficial effects may arise from antioxidant properties that reduce exercise-induced oxidative damage [61] and from the protection of mitochondrial function and improvement of cardiovascular responses in the heat [62]. The combination of caffeine and Panax ginseng (CAF + PG) had a relatively large effect size (SMD = 0.85), although the confidence interval crossed the null line, indicating a lack of statistical significance. This may be due to the limited number of included studies. Nevertheless, it could still exert synergistic effects through the mechanisms of its components:

(1) Caffeine, as an adenosine receptor antagonist, delays perceived fatigue via central nervous system stimulation [63,64].
(2) Panax ginseng (PG) reduces exercise-induced inflammatory markers (e.g., IL-6) and oxidative stress while maintaining cortisol and DHEA balance, thus delaying fatigue onset and promoting recovery [65].

In hot environments, such combination supplements may be more effective than single-agent use.

Branched-chain amino acids (BCAAs) and creatine showed potential benefits under heat stress, but the effects were not definitive. BCAA (SMD = 0.73) may delay central fatigue by reducing serotonin synthesis [66,67], but the wide confidence interval (–0.19 to 1.65) suggests that the effect may be strongly influenced by exercise intensity and environmental temperature. The enhancement of the phosphagen system by creatine may be partially offset by thermal stress in hot environments [68], resulting in a non-significant effect. More precise supplementation strategies are needed in future research.

The effects of ICE + MENTHOL and polyphenol antioxidants were not significant, although the potential synergistic action between ice and menthol warrants further investigation. Sodium citrate and tyrosine showed effects close to zero, indicating limited efficacy in hot environments [33,35,50,51]. Caffeine (CAF) and taurine + caffeine (TAU + CAF) both exhibited positive but unstable effects, particularly TAU + CAF, which may be limited by several factors, such as (1) dosage ratio, (2) individual sensitivity to heat stress, and (3) the influence of hydration status on pharmacokinetics [42,69].

Taken together, these findings illustrate the necessity of considering P-scores and confidence intervals in tandem: high-ranking probabilities should be weighed against the magnitude and precision of effect estimates before firm conclusions are drawn.

### 4.3. Effects on Subjective Perception

Menthol energy demonstrated the most significant positive effect on perceptual outcomes. This strong effect may stem from the unique cooling sensation of menthol [59,70] combined with energy supplementation, which effectively alleviates thermal discomfort and delays fatigue under heat stress [71]. The CAF + PG combination exhibited a moderate positive trend. Although the confidence interval was wide, this suggests a potential synergistic benefit from caffeine’s central stimulation effects [72] and ginseng’s anti-inflammatory properties [73].

Notably, taurine (TAU) showed a negative trend in this domain—an unexpected result that may be attributed to differences in dosing protocols across studies and warrants further investigation. Cold menthol and polyphenol antioxidants both produced non-significant results. The former may be due to the limited efficacy of sensory cooling without concurrent energy support, indicating that future studies should explore combined supplements [74].

In terms of electrolyte supplementation, in contrast to previous findings [41], both high-sodium (High Na^+^) and low-sodium (Low Na^+^) supplements showed opposing but non-significant trends. This reflects the complexity of sodium supplementation strategies and may be influenced by

(1) Individual differences in sweat sodium concentration;
(2) The timing of sodium intake (pre-, during, or post-exercise) affecting plasma volume differently [75,76]

The neutral results observed for creatine and tyrosine suggest that their efficacy in hot environments may be attenuated by heat-related physiological stress [77,78]. Sodium citrate also did not show significant improvement in subjective perception.

Taken together, these results indicate that certain supplements exhibit clear positive effects on endurance and perception in the heat, while others show minimal effects when used alone but perform better in combination—such as caffeine + ginseng. Therefore, future research should further investigate the effects of supplement combinations to optimize endurance exercise performance under heat stress. More studies are also needed to validate these findings.

These discrepancies reinforce that P-scores serve as probabilistic rankings, not standalone indicators of effect. Reliable interpretation, therefore, demands an integrated appraisal of both metrics, overlooking either dimension risks overstating or understating the true impact of a given intervention.

### 4.4. Limitations

The overall robustness of our findings is supported by multiple diagnostics.

(i) The design-by-treatment and node-splitting tests detected no significant global or local inconsistency (all *p* > 0.05), suggesting agreement between direct and indirect evidence. (ii) Bayesian trace plots and PSRF values (~1.00) confirmed satisfactory chain convergence, indicating model stability. (iii) Funnel plots were largely symmetrical, and Egger’s tests were non-significant (endurance *p* = 0.26; perception *p* = 0.94), pointing to a low risk of small-study publication bias.

Taken together, these diagnostics strengthen confidence in the pooled estimates; nevertheless, several caveats remain.

First, the evidence base is thin for certain supplements—some effects (e.g., menthol) rely almost entirely on indirect comparisons, and moderate-to-high heterogeneity persists for these agents, which tempers certainty.

Second, single-ingredient caffeine and nitrate were excluded to preserve network coherence; consequently, our conclusions do not apply to these widely used ergogenic aids. Future NMAs built specifically around caffeine or nitrate will be needed once head-to-head data accumulate.

Third, although global and local inconsistency tests were non-significant, full transitivity cannot be assumed because trials differed in training status, heat acclimation, ambient humidity, and outcome definitions—factors that may bias indirect contrasts.

Fourth, dose–response modeling was impossible: dosing units, loading schedules, and timing relative to exercise were reported inconsistently. Future RCTs should standardize these elements.

Finally, neither funnel-plot symmetry nor Egger tests indicated small-study effects, yet the low number of trials per supplement (<10) limits statistical power; residual publication bias, especially for sparsely studied agents, cannot, therefore, be ruled out. Additionally, the included data did not examine sex-specific differences in bioavailability. Only 9.2% (n = 60) of participants in the included studies were female, so extrapolation of results to female athletes should be performed with caution.

Considering these factors, it is reasonable to conclude that while this network meta-analysis has limitations, it nonetheless provides valuable and important evidence for the use of nutritional supplementation in hot environments.

To further ensure the validity of indirect comparisons, we assessed the transitivity assumption by examining potential effect modifiers such as participant demographics, intervention types, environmental conditions, and outcome definitions. No substantial clinical or methodological heterogeneity was identified, but we acknowledge that some network nodes remain sparsely connected and encourage cautious interpretation where direct evidence is lacking.

## 5. Conclusions

Overall, (i) in terms of endurance, menthol and taurine demonstrated relatively clear performance-enhancing effects under high-temperature conditions, whereas the effects of other supplements still require further validation through high-quality studies. (ii) From the perspective of subjective perception, menthol energy gel exhibited the most definitive potential for improving exercise performance in the heat, while the effects of other supplements showed considerable heterogeneity.

Therefore, future research should more strictly control environmental conditions, supplementation protocols, and participant characteristics to more accurately assess the actual efficacy of each supplement under hot conditions.

Further high-quality studies are needed to explore the effects of various nutritional supplements on athletes’ performance in high-temperature environments and to identify those that are most suitable for application in endurance sports under heat stress.

This will facilitate scientific decision-making for coaches, athletes, and researchers in developing personalized, heat-adaptive, and nutritional strategies, ultimately helping athletes better improve their athletic performance in hot environments.

## Figures and Tables

**Table 1:** Characteristics of the 22 RCTs included in the network meta-analysis.

Study Information	Sample Size	Age	Gender (% male)	Special	Supplement Type, Dose	Time	Temperature, Relative Humidity	Outcome	Performance Testing	Result
(Author;Year; Country/Region)	Exp: n = XX; Ctrl: n = XX	XX.X ± X.X years	XX.X%	[special] (e.g., 1500 m, competitive walking)	[supplement] (e.g., tyrosine, creatine)	every 15 min during exercise	30 °C; RH, 50%	EE, SP	30 min cycle at 55% VO_2_max	
Kilduff et al. 2004 UK [29]	Exp: n = 11; Ctrl: n = 10	27 ± 4 years	100.00%	endurance training|	Creatine (20 g/day Cr + 140 g/day glucose polymer)	7 days before test	30.3 °C	EE, SP	Constant-load exercise to exhaustion at 63% VO_2_max	Time to exhaustion ↔, RPE ↓
Wright et al. 2007 USA [30]	Exp: n = 10; Ctrl: n = 10	25.7 ± 4.9 years	100.00%	cycling	Creatine (20 g·d^−1^ Cr + 140 g·d^−1^ glucose polymer)	once/day before session	35 °C; RH 60%	EE, SP	Cycling to exhaustion at 63 ± 5% VO_2_max	Time to exhaustion ↔, Peak power ↑, Mean power ↑ RPE ↓, thermal comfort ↑
Hadjicharalambous et al. 2008 UK [31]	Exp: n = 11; Ctrl: n = 10	27 ± 4 years	100.00%	running	Creatine (20 g·d^−1^ Cr + 140 g·d^−1^ glucose polymer)	once/day before session	30.3 °C; RH 70%	EE, SP	Running to exhaustion at 63 ± 5% VO_2_max	Time to exhaustion ↔, Peak power ↑, Mean power ↑ RPE ↓, thermal comfort ↑
Volek et al. 2001 USA [32]	Exp: n = 10; Ctrl: n = 10	23.0 ± 1.0 years	100.00%	cycling	Creatine (0.3 g·kg^−1^ body weight)	every 2–3 h, divided into 5 doses	37 °C; RH 80%	EE, SP	30 min cycling at 60–70% VO_2_peak + 3 × 10 s sprints	Sprint performance ↑
Tumilty et al. 2011 UK [33]	Exp: n = 8; Ctrl: n = 8	32 ± 11 years	100.00%	team and endurance sports	Tyrosine (150 mg/kg body mass)	1 h before exercise	30 °C, RH 60%	EE, SP	Cycling to exhaustion at 68 ± 5% VO_2_peak	RPE ↔, Thermal sensation ↔ Time to exhaustion ↑
Tumilty et al. 2014 UK [34]	Exp: n = 7; Ctrl: n = 7	30 ± 6 years	100.00%	cycling	Tyrosine (151 mg/kg body mass)	Once, 1 h before exercise	30 °C, RH 60%	EE, SP	60 min cycling at 57% ± 4% VO_2_peak followed by a time trial	Time trial performance ↔ RPE ↔, Thermal sensation ↔
Watson et al. 2012 UK [35]	Exp: n = 10; Ctrl: n = 10	23 ± 3 years	100.00%	cycling	Tyrosine(150 mg/kg BM)	once/day before session	30.2 °C; RH 50%	EE, SP	Cycling to exhaustion at 69% ± 3% VO_2_max	Time to exhaustion ↔RPE ↔, thermal comfort ↔
Tran Trong et al. 2015 France [36]	Exp: n = 10; Ctrl: n = 10	41 ± 17 years	100.00%	cycling, running	I-SM(190 mL of beverage with 0.05 mL menthol)	every 15 min during exercise	27.6 °C; RH 57%	EE, SP	5 blocks of 4-km cycling and 1.5-km running	Time to exhaustion ↓ RPE ↔, thermal comfort ↓
Stevens et al. 2015 Australia [37]	Exp: n = 11; Ctrl: n = 11	29 ± 9 years	100.00%	running	I-SM rinse(Ice slurry: 7.5 g/kg BM; Menthol rinse: 0.01% solution)	Ice slurry: once/day before session; Menthol rinse: every 1 km during exercise	33 °C; RH 46%	EE, SP	5-km running time trial	Time to exhaustion ↓ (Menthol rinse)RPE ↔, thermal comfort ↓ (Menthol rinse)
Vogel et al. 2022 Australia [38]	Exp: n = 27; Ctrl: n = 27	34.8 ± 6.7 years	74.00%	endurance sports	Menthol (0.1%, 0.3%, 0.5%, 0.7%)	once before exercise	35 °C, RH 65%	EE	45 min running/racewalking	Cooling sensation ↑, Irritation ↑
Hamouti et al. 2012 Spain [39]	Exp: n = 10; Ctrl: n = 10	33 ± 6 years	100.00%	cycle	SW (82–164 mM Na^+^)	90 min before exercise	33 °C, RH 30%	EE, SP	120 min cycling at 63% VO_2_max + time trial	Time-trial performance ↑, Heart rate ↓, Stroke volume ↓, RPE ↓
Sims et al. 2007 New Zealand [40])	Exp: n = 13; Ctrl: n = 13	26 ± 6 years	100.00%	cycle	Sodium (164 mmol Na^+^/L)	105 min before exercise	32 °C, RH 50%	EE, SP	Cycling to exhaustion at 70% VO_2_peak	Time to exhaustion ↑, Core temperature rise ↓, RPE ↓
Sims et al. 2007 New Zealand [41]	Exp: n = 8; Ctrl: n = 8	36 ± 11 years	100.00%	running	H-SB (10 mL/kg body mass, 164 mmol/L Na^+^ (High Na^+^); 10 mmol/L Na^+^ (Low Na^+^))	Ingested in seven portions across 60 min, beginning 105 min before exercise	32 °C, RH 50%	EE, SP	Running to exhaustion at 70% VO_2_max	Time to exhaustion ↑, Perceived exertion ↓
Yu et al. 2024 China [42]	Exp: n = 12; Ctrl: n = 12	23.75 ± 2.41 years	100.00%	NP	C + T (5 mg/kg CAF + 50 mg/kg TAU)	once 1 h before exercise	35 °C, RH 65%	EE, SP	Time to exhaustion cycling at ventilatory threshold	Time to exhaustion ↑, Blood lactate ↓Core temperature ↓
Bandyopadhyay et al. 2011 India [43]	Exp: n = 9; Ctrl: n = 9	25.4 ± 6.9 years	100.00%	running	C + PG(5 mg/kg BW caffeine + 200 mg Panax ginseng)	Once, 1 h before exercise	31 °C; RH 70%	EE, SP	Running to exhaustion at 70% VO_2_max	Time to exhaustion ↑RPE ↔
Ping et al. 2011 Indian [44]	Exp: n = 9; Ctrl: n = 9	25.4 ± 6.9 years	100.00%	running	PG (200 mg)	once/day before session	31 °C; RH 70%	EE, SP	Running to exhaustion at 70% VO_2_max	Time to exhaustion ↔ RPE ↔, thermal comfort ↔
Watson et al. 2004 UK [45]	Exp: n = 8; Ctrl: n = 8	28.5 ± 8.2 years	100.00%	cycling	BCAA (12 g/L BCAA solution)	every 30 min before exercise, every 15 min during exercise	30.0 °C; RH 38%	EE, SP	Cycling to exhaustion at 50% VO_2_peak	Time to exhaustion ↔
Mittleman et al. 1998 USA [46]	Exp: n = 8; Ctrl: n = 8	Not Reported	50.00%	NP	BCAA	NP	40 °C	EE, SP	Cycling to exhaustion at 40% VO_2_peak	Time to exhaustion ↑, RPE ↓
Cathcart et al. 2011 UK [47]	Exp (CHO): n = 13; Ctrl (CHO-PRO): n = 10	32 ± 1 years	83.3%	mountain bike	CHO, CHO–PRO (76 g/L CHO, 18 g/LPRO + 72 g/LCHO)	once/day	33 °C; RH, 42%	EE, SP	Cycling to exhaustion at VO_2_peak	Time to complete race ↓, Muscle soreness ↔
Easton et al. 2007 UK [48]	Exp: n = 12; Ctrl: n = 11	33 ± 6 years	100.00%	cycle	CG (11.4 g Cr + 1 g Gly/kg body mass)	Twice daily for 7 days	30 °C; RH 70%	EE, SP	40 min cycling at 63% WRmax + 16.1 km time trial	Heart rate ↓, Rectal temperature ↓, RPE ↓
Page et al. 2019 UK [49]	Exp: n = 11; Ctrl: n = 11	23 ± 2 years	100.00%	cycle	Taurine (50 mg/kg body mass)	2 h before exercise	35 °C, RH 40%	EE, SP	Cycling to exhaustion at ventilatory threshold	Time to exhaustion ↑, Sweat rate ↑, Core temperature ↓, RPE ↓
Trinity et al. 2014 USA [50]	Exp: n = 12; Ctrl: n = 12	26.8 ± 5.0 years	100.00%	cycling	PA (1800-ppm polyphenols, PE)	Twice daily for 7 days, last dose 30 min before exercise	31.5 °C, RH 55%	EE, SP	10 min time trial following 50 min of moderate intensity cycling	Time trial performance ↔, Time to fatigue ↔ RPE ↔
Vaher et al. 2015 Estonia [51]	Exp: n = 16; Ctrl: n = 16	25.8 ± 4.4 years	100.00%	running	SC (500 mg/kg BM)	once/day before session	32 °C; RH 50%	EE, SP	5000-m running time trial	Time to exhaustion ↔RPE ↓, thermal comfort ↔
Fleischmann et al. 2019 Israel [52]	Exp: n = 12; Ctrl: n = 10	23.14 ± 3.5 years	100.00%	military	Astaxanthin (12 mg/day)	once/day for 30 days	40 °C, RH 40%	EE	VO_2_max test and 2 h walk at 40 °C, 40% RH	Blood lactate ↓, End recovery VO_2_ ↓
Kajiki et al. 2024 Japan [53]	Exp: n = 12; Ctrl: n = 12	25 ± 5 years	58.00%	NP	CW (150 mL for males, 100 mL for females)	20 and 40 min post-exercise	35 °C, RH 50%	EE, SP	60 min cycling at 45% peak oxygen uptake	Mean arterial pressure ↑, Cerebral blood flow index ↑Mouth exhilaration ↑, Sleepiness ↓

BCAA: branched-chain amino acid, PA: polyphenol antioxidants, PE: primary ellagitannins, PG: panax ginseng, SC: sodium citrate; EE: exercise endurance, SP:subjective peception; NP: Not Reported; CW:Carbonated water; CG:creatine Glycerol; H-SB: High-sodium beverage; I-S/M: Ice-slurry/Menthol; C + T: Caffeine + Taurine; C + PG: Caffeine + Panax ginseng; SW: Sodium + Water. the arrow symbols ↑, ↓, and ↔ represent a significant increase, a sig-nificant decrease, and no significant change in the indicator after the supplement intervention, respectively. The specific effects need to be interpreted in conjunction with the attributes of the indicator itself (e.g., an increase in endurance is beneficial for ↑, and a decrease in core body temperature is beneficial for ↓).

## Supplementary Materials

The following supporting information can be downloaded at: https://www.mdpi.com/article/10.3390/nu17132141/s1, Supplement Table S1: Search strategy of PubMed, Embase, Cochrane, Web of Science, and EBSCO; Supplement Table S2: Characteristics of the included studies; Supplement Figure S1: Risk of bias assessment; Supplement Figure S2: The network graph illustrates the effects of various nutritional supplements on Endurance Performance and Subjective Perception; Supplement Figure S3: Funnel plots were constructed for different nutritional supplements in the pairwise meta-analysis; Supplement Figure S4: Iterative and Posterior Distribution Maps of Endurance Performance and Subjective Perception; Supplement Figure S5: Forest plots of eligible comparisons of Endurance Performance and Subjective Perception; Supplement Figure S6: Area under the curve for cumulative ranking probability of each intervention on Endurance Performance and Subjective Perception; Supplement S9: R language source code.
