# Supplementary material for: Effects of Nutritional Supplements on Endurance Performance and Subjective Perception in Athletes Exercising in the Heat: A Systematic Review and Network Meta-Analysis
Source: Nutrients. 2025 Jun 27;17(13):2141. doi: 10.3390/nu17132141 (PMC12252039; doi:10.3390/nu17132141)
Supplement: Supplementary file 1 [file nutrients-17-02141-s001.zip › nutrients-3697041-supplementary.pdf]

Supplementary materials	Page
**Supplement Table S1.** Search strategy of PubMed, Embase, Cochrane, Web of Science, and EBSCO.	2-3
**Supplement Table S2.** Characteristics of the included studies.	4-8
**Supplement Figure S1.** Risk of bias assessment.	9-10
**Supplement Figure S2.** The network graph illustrates the effects of various nutritional supplements on Endurance Performance and Subjective Perception.	11-12
**Supplement Figure S3.** Funnel plots were constructed for different nutritional supplements in the pairwise meta-analysis.	13-14
**Supplement Figure S4.** Iterative and Posterior Distribution Maps of Endurance Performance and Subjective Perception.	15-16
**Supplement Figure S5.** Forest plots of eligible comparisons of Endurance Performance and Subjective Perception.	17
**Supplement Figure S6.** Area under the curve for cumulative ranking probability of each intervention on Endurance Performance and Subjective Perception.	18-19
**Supplement S9.** R language source code	20

Supplement Table S1. Search strategy of PubMed, Embase, Cochrane, Web of Science, and EBSCO.

Database	Search Terms		
	Sport	Dietary supplements	Exercise performance
PubMed [Title/Abstract]	Athletes[MeSH Terms] OR "sport" OR "endurance athletes" OR "team sport athletes" OR "football players" OR "exercise"	"dietary supplements"[MeSH Terms] OR "nutritional supplements" OR "creatine" OR "branched-chain amino acids" OR BCAA OR "beetroot juice" OR "tyrosine" OR "beta-alanine" OR "electrolytes" OR "carbohydrate" OR "carbohydrate supplementation" OR "protein" OR "protein supplementation" OR "cherry juice" OR "sour cherry juice" OR "sodium bicarbonate" OR "vitamins" OR "vitamin supplementation" OR "taurine" OR "medium-chain triglycerides" OR MCT OR "probiotics" OR "multi-ingredient supplements"	"endurance exercise performance"[MeSH Terms] OR "exercise performance" OR "physical endurance" OR "endurance capacity" OR "aerobic performance" OR "time to exhaustion" OR "time trial" OR "running distance" OR "sprint performance" OR "power output" OR "perceived exertion" OR "rating of perceived exertion" OR RPE OR "fatigue perception" OR "subjective fatigue" OR "thermal sensation" OR "thermal perception" OR "subjective recovery" OR "recovery perception" OR "perceptual responses"
Embase [Title/Abstract]	Athletes [Emtree term] OR sport OR endurance athletes OR team sport athletes OR football players OR exercise	Dietary supplements[Emtree term] OR nutritional supplements OR creatine OR branched-chain amino acids OR BCAA OR beetroot juice OR tyrosine OR beta-alanine OR electrolytes OR carbohydrate OR carbohydrate supplementation OR protein OR protein supplementation OR cherry juice OR sour cherry juice OR sodium bicarbonate OR	Endurance exercise performance[Emtree term] OR exercise performance OR physical endurance OR endurance capacity OR aerobic performance OR time to exhaustion OR time trial OR running distance OR sprint performance OR power output"OR perceived exertion OR rating of perceived exertion OR RPE OR fatigue perception OR subjective fatigue OR thermal sensation OR thermal perception OR subjective

		vitamins OR vitamin supplementation OR taurine OR medium-chain triglycerides OR MCT OR probiotics OR multi-ingredient supplements	recovery OR recovery perception OR perceptual responses
Cochrane [Title/Abstract/keywords]	Athletes [MeSH Terms] OR sport OR endurance athletes OR team sport athletes OR football players OR exercise	Dietary supplements[MeSH Terms] OR nutritional supplements OR creatine OR branched-chain amino acids OR BCAA OR beetroot juice OR tyrosine OR beta-alanine OR electrolytes OR carbohydrate OR carbohydrate supplementation OR protein OR protein supplementation OR cherry juice OR sour cherry juice OR sodium bicarbonate OR vitamins OR vitamin supplementation OR taurine OR medium-chain triglycerides OR MCT OR probiotics OR multi-ingredient supplements	Endurance exercise performance[MeSH Terms] OR exercise performance OR physical endurance OR endurance capacity OR aerobic performance OR time to exhaustion OR time trial OR running distance OR sprint performance OR power output"OR perceived exertion OR rating of perceived exertion OR RPE OR fatigue perception OR subjective fatigue OR thermal sensation OR thermal perception OR subjective recovery OR recovery perception OR perceptual responses
Web of Science	athletes OR sport OR endurance athletes OR team sport athletes OR football players OR exercise	dietary supplements OR nutritional supplements OR creatine OR branched-chain amino acids OR BCAA OR beetroot juice OR tyrosine OR beta-alanine OR electrolytes OR carbohydrate OR carbohydrate supplementation OR protein OR protein supplementation OR cherry juice OR sour cherry juice OR sodium bicarbonate OR vitamins OR vitamin supplementation OR	endurance exercise performance OR exercise performance OR physical endurance OR endurance capacity OR aerobic performance OR time to exhaustion OR time trial OR running distance OR sprint performance OR power output"OR perceived exertion OR rating of perceived exertion OR RPE OR fatigue perception OR subjective fatigue OR thermal sensation OR thermal perception OR subjective recovery OR recovery

		taurine OR medium-chain triglycerides OR MCT OR probiotics OR multi-ingredient supplements	perception OR perceptual responses
EBSCO [Abstract]	athletes OR sport OR endurance athletes OR team sport athletes OR football players OR exercise	dietary supplements OR nutritional supplements OR creatine OR branched-chain amino acids OR BCAA OR beetroot juice OR tyrosine OR beta-alanine OR electrolytes OR carbohydrate OR carbohydrate supplementation OR protein OR protein supplementation OR cherry juice OR sour cherry juice OR sodium bicarbonate OR vitamins OR vitamin supplementation OR taurine OR medium-chain triglycerides OR MCT OR probiotics OR multi-ingredient supplements	endurance exercise performance OR exercise performance OR physical endurance OR endurance capacity OR aerobic performance OR time to exhaustion OR time trial OR running distance OR sprint performance OR power output"OR perceived exertion OR rating of perceived exertion OR RPE OR fatigue perception OR subjective fatigue OR thermal sensation OR thermal perception OR subjective recovery OR recovery perception OR perceptual responses

Supplement Table S2. Characteristics of the included studies.

Study Information	Sample Size	Age	Gender (% male)	Special	Supplement type,dose	Time	temperature，relative humidity	Outcome	Performance Testing	Result
(Author;Year; Country/Region)	Exp: n=XX; Ctrl: n=XX	XX.X ± X.X years	XX.X%	[special] (e.g., 1500m，competitive walking)	[supplement] (e.g., tyrosine，creatine)	every 15 min during exercise	30 ℃;RH,50%	EE,SP	30 min cycle at 55% VO2max	
Kilduff et al. 2004 UK(1)	Exp: n=11; Ctrl: n=10	27±4 years	100.00%	endurance training	Creatine（20g/day Cr + 140g/day glucose polymer）	7 days before test	30.3°C	EE,SP	Constant-load exercise to exhaustion at 63% VO_2max	Time to exhaustion ↔，RPE ↓
Wright et al. 2007 USA(2)	Exp: n=10; Ctrl: n=10	25.7±4.9 years	100.00%	cycling	Creatine(20 g·d⁻¹ Cr + 140 g·d⁻¹ glucose polymer)	once/day before session	35°C; RH 60%	EE,SP	Cycling to exhaustion at 63±5% VO_2max	Time to exhaustion ↔, Peak power ↑, Mean power ↑ RPE ↓, thermal comfort ↑
Hadjicharalambous et al. 2008 UK(3)	Exp: n=11; Ctrl: n=10	27±4 years	100.00%	running	Creatine(20 g·d⁻¹ Cr + 140 g·d⁻¹ glucose polymer)	once/day before session	30.3°C; RH 70%	EE,SP	Running to exhaustion at 63±5% VO_2max	Time to exhaustion ↔, Peak power ↑, Mean power ↑ RPE ↓, thermal comfort ↑
Volek et al. 2001 USA(4)	Exp: n=10; Ctrl: n=10	23.0±1.0 years	100.00%	cycling	Creatine（0.3 g·kg⁻¹ body weight）	every 2-3 h, divided into 5 doses	37°C; RH 80%	EE,SP	30 min cycling at 60-70% VO_2peak + 3×10 s sprints	Sprint performance ↑
Tumilty et al.2011 UK(5)	Exp: n=8; Ctrl: n=8	32±11 years	100.00%	team and endurance sports	Tyrosine （150 mg/kg body mass）	1 hour before exercise	30°C, RH 60%	EE,SP	Cycling to exhaustion at 68±5% VO_2peak	RPE ↔, Thermal sensation ↔ Time to exhaustion ↑
Tumilty et al.2014 UK(6)	Exp: n=7; Ctrl: n=7	30±6 years	100.00%	cycling	Tyrosine （151 mg/kg body mass）	Once, 1 hour before exercise	30°C, RH 60%	EE,SP	60 min cycling at 57% ± 4% VO_2peak followed by a time trial	Time trial performance ↔ RPE ↔, Thermal sensation ↔
Watson et al. 2012 UK(7)	Exp: n=10; Ctrl: n=10	23±3 years	100.00%	cycling	Tyrosine(150 mg/kg BM)	once/day before session	30.2°C; RH 50%	EE,SP	Cycling to exhaustion at 69% ± 3% VO_2max	Time to exhaustion ↔RPE ↔, thermal comfort ↔
Tran Trong et al. 2015 France(8)	Exp: n=10; Ctrl: n=10	41±17 years	100.00%	cycling, running	I-SM(190 ml of beverage with 0.05 mL menthol)	every 15 min during exercise	27.6°C; RH 57%	EE,SP	5 blocks of 4-km cycling and 1.5-km running	Time to exhaustion ↓ RPE ↔, thermal comfort ↓
Stevens et al. 2015 Australia(9)	Exp: n=11; Ctrl: n=11	29±9 years	100.00%	running	I-SM rinse(Ice slurry: 7.5 g/kg BM; Menthol rinse: 0.01% solution)	Ice slurry: once/day before session; Menthol rinse: every 1 km during exercise	33°C; RH 46%	EE,SP	5-km running time trial	Time to exhaustion ↓ (Menthol rinse)RPE ↔, thermal comfort ↓ (Menthol rinse)
Vogel et al. 2022 Australia(10)	Exp: n=27; Ctrl: n=27	34.8±6.7 years	74.00%	endurance sports	Menthol(0.1%, 0.3%, 0.5%, 0.7%)	once before exercise	35°C, RH 65%	EE	45min running/racewalking	Cooling sensation ↑, Irritation ↑

Hamouti et al. 2012 Spain(11)	Exp: n=10; Ctrl: n=10	33±6years	100.00%	cycle	SW（82-164 mM Na+）	90 min before exercise	33°C, RH 30%	EE,SP	120 min cycling at 63% VO_2max + time trial	Time-trial performance ↑, Heart rate ↓, Stroke volume ↓，RPE ↓
Sims et al. 2007 New Zealand(12)	Exp: n=13; Ctrl: n=13	26±6 years	100.00%	cycle	Sodium（164 mmol Na+/L）	105 min before exercise	32°C, RH 50%	EE,SP	Cycling to exhaustion at 70% VO_2peak	Time to exhaustion ↑, Core temperature rise ↓，RPE ↓
Sims et al. 2007 New Zealand(13)	Exp: n=8; Ctrl: n=8	36±11 years	100.00%	running	H-SB（10mL/kg body mass, 164 mmol/L Na+ (High Na+); 10 mmol/L Na+ (Low Na+) ）	Ingested in seven portions across 60 min, beginning 105 min before exercise	32°C, RH 50%	EE,SP	Running to exhaustion at 70% VO_2max	Time to exhaustion ↑，Perceived exertion ↓
Yu et al. 2024 China(14)	Exp: n=12; Ctrl: n=12	23.75±2.41 years	100.00%	NP	C+T(5 mg/kg CAF + 50 mg/kg TAU)	once 1 hour before exercise	35°C, RH 65%	EE,SP	Time to exhaustion cycling at ventilatory threshold	Time to exhaustion ↑, Blood lactate ↓Core temperature ↓
Bandyopadhyay et al. 2011 India(15)	Exp: n=9; Ctrl: n=9	25.4±6.9 years	100.00%	running	C+PG(5 mg/kg BW caffeine + 200 mg Panax ginseng)	Once, 1 hour before exercise	31°C; RH 70%	EE,SP	Running to exhaustion at 70% VO_2max	Time to exhaustion ↑RPE ↔
Wong et al. 2011 Malaysia(16)	Exp: n=9; Ctrl: n=9	25.4±6.9 years	100.00%	running	PG(200 mg)	once/day before session	31°C; RH 70%	EE,SP	Running to exhaustion at 70% VO_2max	Time to exhaustion ↔ RPE ↔, thermal comfort ↔
Watson et al. 2004 UK(17)	Exp: n=8; Ctrl: n=8	28.5±8.2 years	100.00%	cycling	BCAA(12 g/L BCAA solution)	every 30 min before exercise, every 15 min during exercise	30.0°C; RH 38%	EE,SP	Cycling to exhaustion at 50% VO_2peak	Time to exhaustion ↔
Mittleman et al. 1998 USA(18)	Exp: n=8; Ctrl: n=8	Not Reported	50.00%	NP	BCAA	NP	40°C	EE,SP	Cycling to exhaustion at 40% VO_2peak	Time to exhaustion ↑，RPE ↓
Cathcart et al. 2011 UK(19)	Exp（CHO）: n=13; Ctrl（CHO-PRO）: n=10	32±1years	83.3%	mountain bike	CHO,CHO–PRO（76g/L CHO,18g/LPRO+72g/LCHO）	once/day	33 °C;RH,42%	EE,SP	Cycling to exhaustion at VO2peak	Time to complete race ↓，Muscle soreness ↔
Easton et al. 2007 UK(20)	Exp: n=12; Ctrl: n=11	33±6years	100.00%	cycle	CG（11.4g Cr + 1g Gly/kg body mass ）	Twice daily for 7 days	30°C; RH 70%	EE,SP	40min cycling at 63% WRmax + 16.1km time trial	Heart rate ↓, Rectal temperature ↓，RPE ↓
Page et al. 2019 UK(21)	Exp: n=11; Ctrl: n=11	23±2 years	100.00%	cycle	Taurine（50 mg/kg body mass）	2 hours before exercise	35°C, RH 40%	EE,SP	Cycling to exhaustion at ventilatory threshold	Time to exhaustion ↑, Sweat rate ↑, Core temperature ↓，RPE ↓
Trinity et al.2014 USA(22)	Exp: n=12; Ctrl: n=12	26.8±5.0 years	100.00%	cycling	PA（1800-ppm polyphenols, PE）	Twice daily for 7 days, last dose 30 min before exercise	31.5°C, RH 55%	EE,SP	10 min time trial following 50 min of moderate intensity cycling	Time trial performance ↔, Time to fatigue ↔ RPE ↔

Vaher et al. 2015 Estonia(23)	Exp: n=16; Ctrl: n=16	25.8±4.4 years	100.00%	running	SC(500 mg/kg BM)	once/day before session	32°C; RH 50%	EE,SP	5,000-m running time trial	Time to exhaustion ↔RPE ↓, thermal comfort ↔
Fleischman n et al. 2019 Israel(24)	Exp: n=12; Ctrl: n=10	23.14±3.5 years	100.00%	military	Astaxanthin(12 mg/day)	once/day for 30 days	40°C, RH 40%	EE	VO_2max test and 2h walk at 40°C, 40% RH	Blood lactate ↓, End recovery VO_2 ↓
Kajiki et al. 2024 Japan(25)	Exp: n=12; Ctrl: n=12	25±5 years	58.00%	NP	CW(150 mL for males, 100 mL for females)	20 and 40 min post-exercise	35°C, RH 50%	EE,SP	60min cycling at 45% peak oxygen uptake	Mean arterial pressure ↑, Cerebral blood flow index ↑Mouth exhilaration ↑, Sleepiness ↓

BCAA:branched-chain amino acid , PA:polyphenol antioxidants , PE:primary ellagitannins , PG:panax ginseng ,SC:sodium citrate; EE:exercise endurance , SP:subjective peception；NP:Not Reported ; CW:Carbonated water; CG:creatine Glycerol; H-SB:High-sodium beverage; I-S/M:Ice-slurry/Menthol;C+T:Caffeine + Taurine; C+PG:Caffeine + Panax ginseng; SW:Sodium+Water

Supplement Figure S1. Risk of bias assessment.

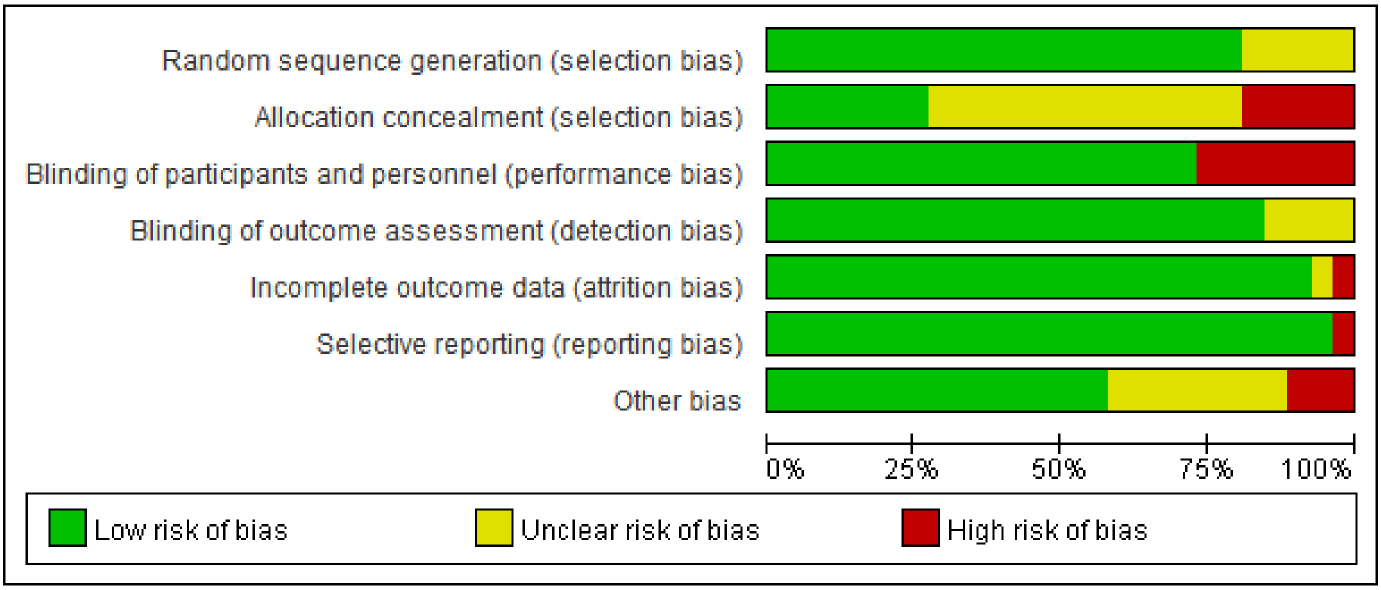

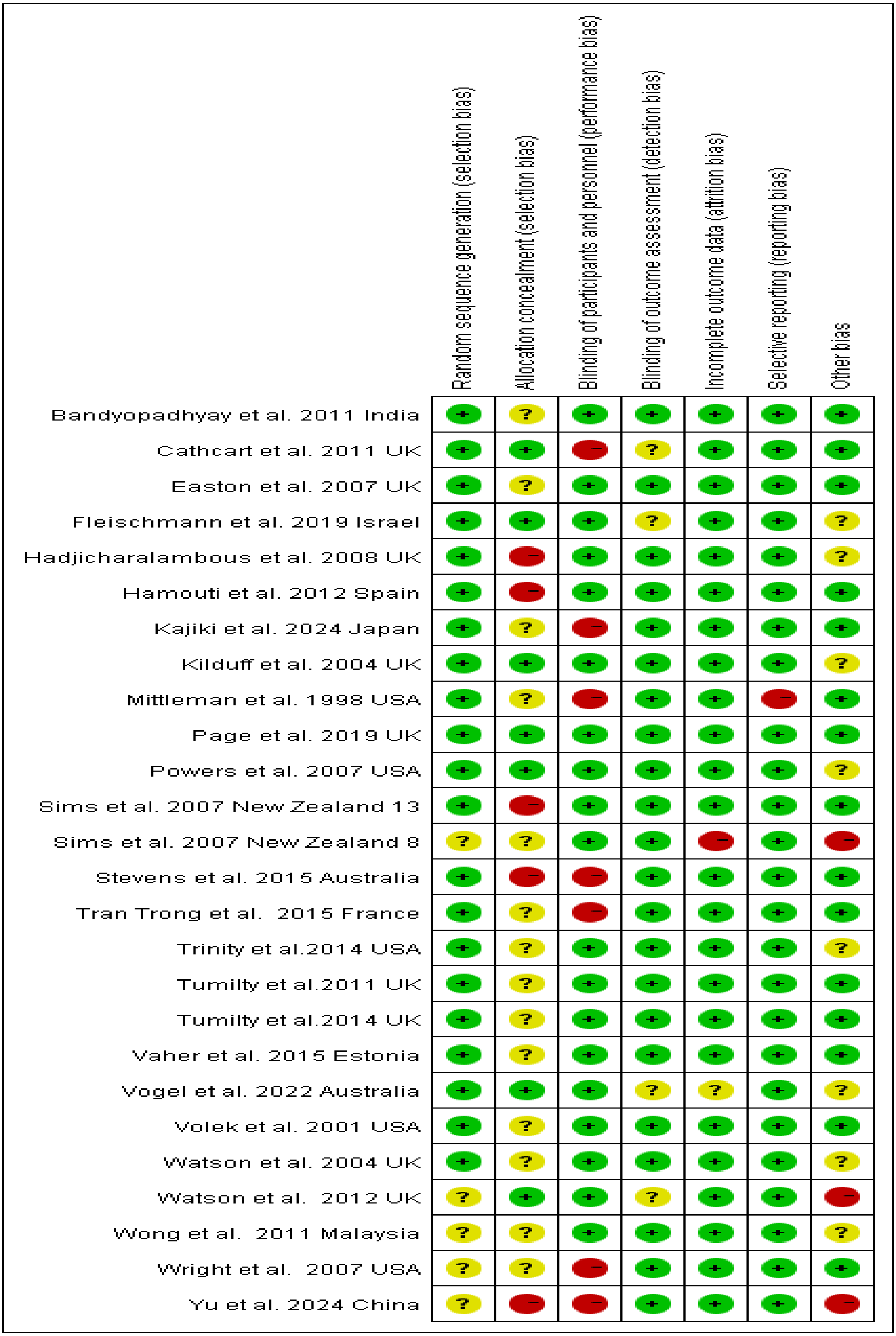
Random sequence generation (selection bias)
Allocation concealment (selection bias)
Blinding of participants and personnel (performance bias)
Blinding of outcome assessment (detection bias)
Incomplete outcome data (attrition bias)
Selective reporting (reporting bias)
Other bias
Bandyopadhyay et al. 2011 India + ? + + + + +
Cathcart et al. 2011 UK + + − ? + + +
Easton et al. 2007 UK + ? + + + + +
Fleischmann et al. 2019 Israel + + + ? + + ?
Hadjicharalambous et al. 2008 UK + − + + + + ?
Hamouti et al. 2012 Spain + − + + + + +
Kajiki et al. 2024 Japan + ? − + + + +
Kilduff et al. 2004 UK + + + + + + ?
Mittleman et al. 1998 USA + ? − + + − +
Page et al. 2019 UK + + + + + + +
Powers et al. 2007 USA + + + + + + ?
Sims et al. 2007 New Zealand 13 + − + + + + +
Sims et al. 2007 New Zealand 8 ? ? + + − + −
Stevens et al. 2015 Australia + − − + + + +
Tran Trong et al. 2015 France + ? − + + + +
Trinity et al.2014 USA + ? + + + + ?
Tumilty et al.2011 UK + ? + + + + +
Tumilty et al.2014 UK + ? + + + + +
Vaher et al. 2015 Estonia + ? + + + + +
Vogel et al. 2022 Australia + + + ? ? + ?
Volek et al. 2001 USA + ? + + + + +
Watson et al. 2004 UK + ? + + + + ?
Watson et al. 2012 UK ? + + ? + + −
Wong et al. 2011 Malaysia ? ? + + + + ?
Wright et al. 2007 USA ? ? − + + + +
Yu et al. 2024 China ? − − + + + −

Supplement Figure S2. The network graph illustrates the effects of various nutritional supplements on Endurance Performance and Subjective Perception.

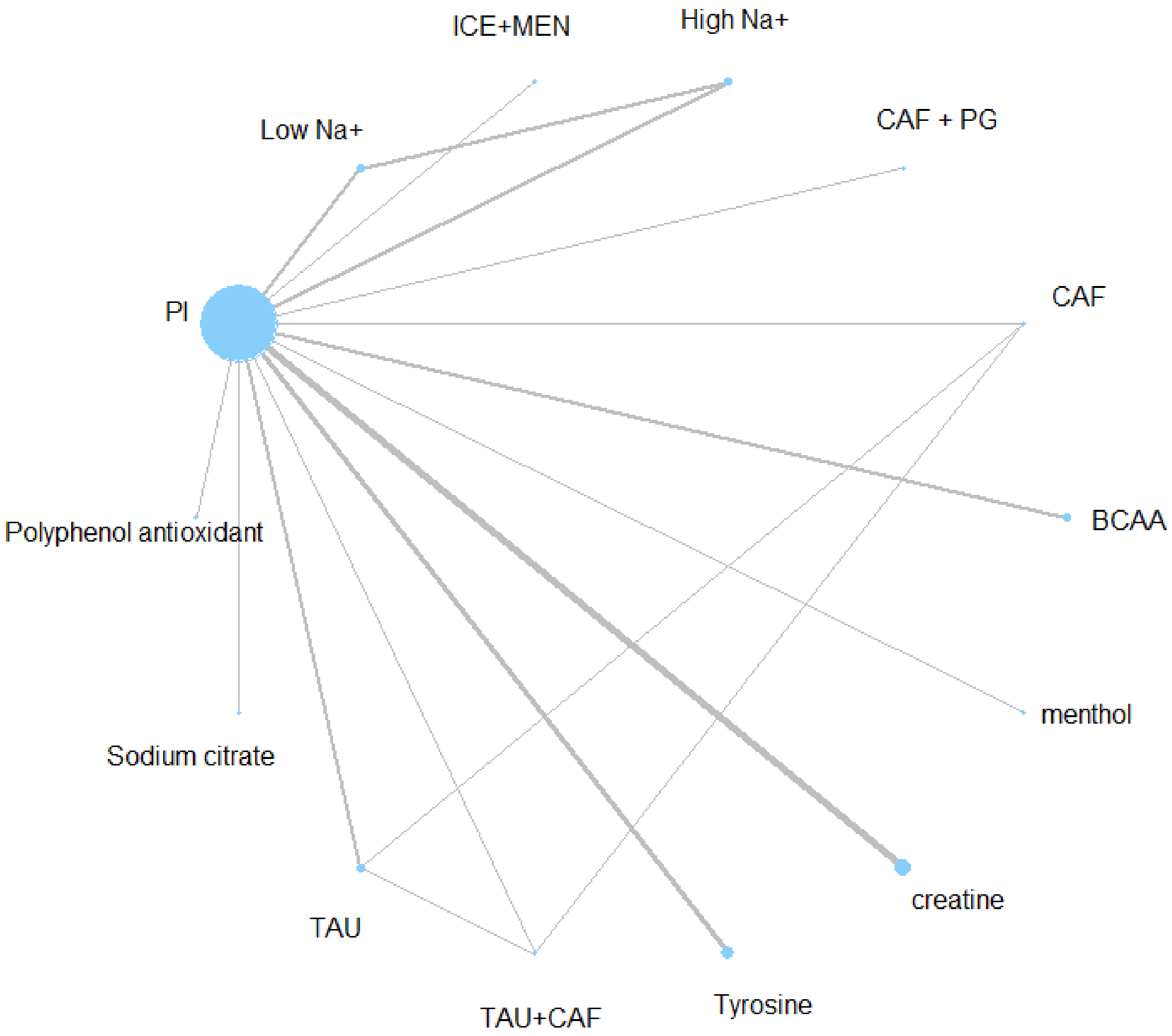

Endurance Performance

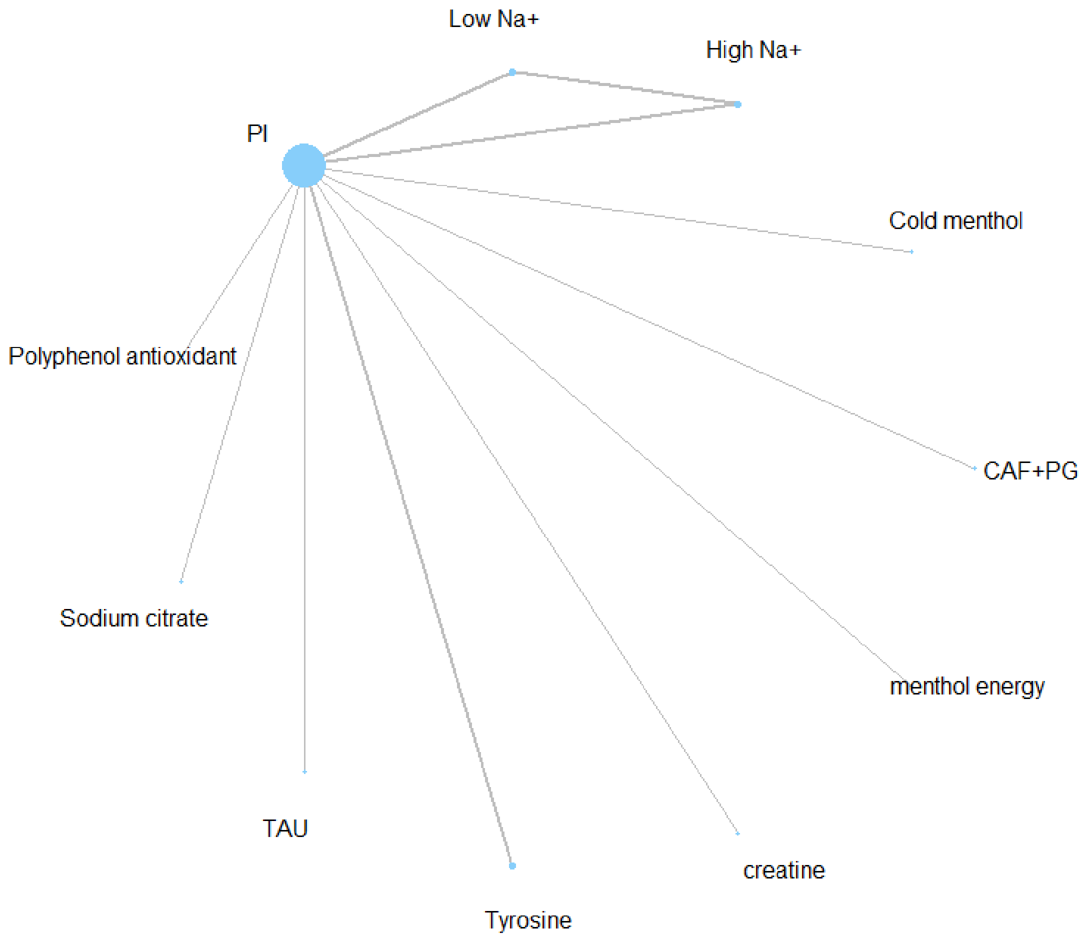

Subjective Perception

Supplement Figure S3. Funnel plots were constructed for different nutritional supplements in the pairwise meta-analysis.

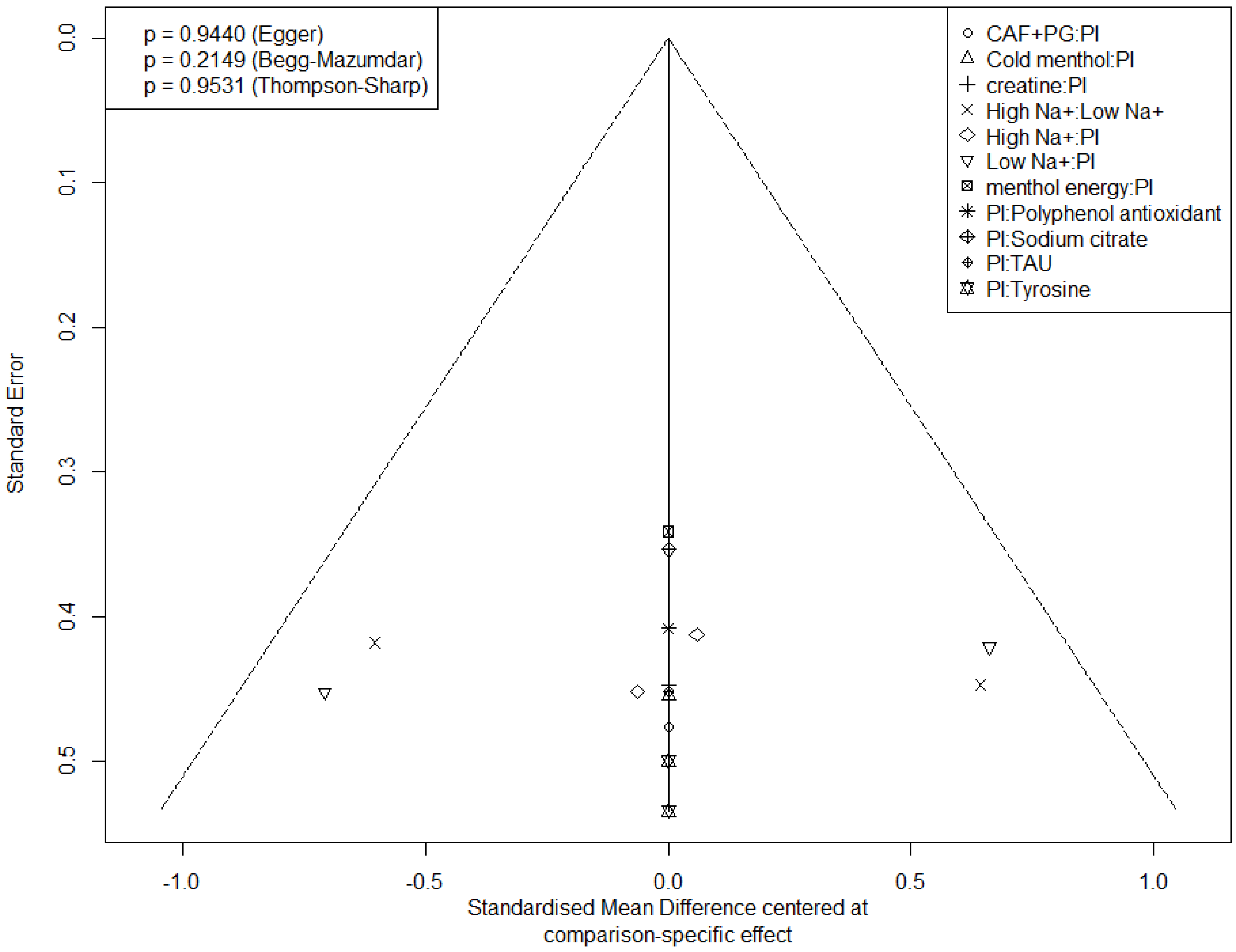

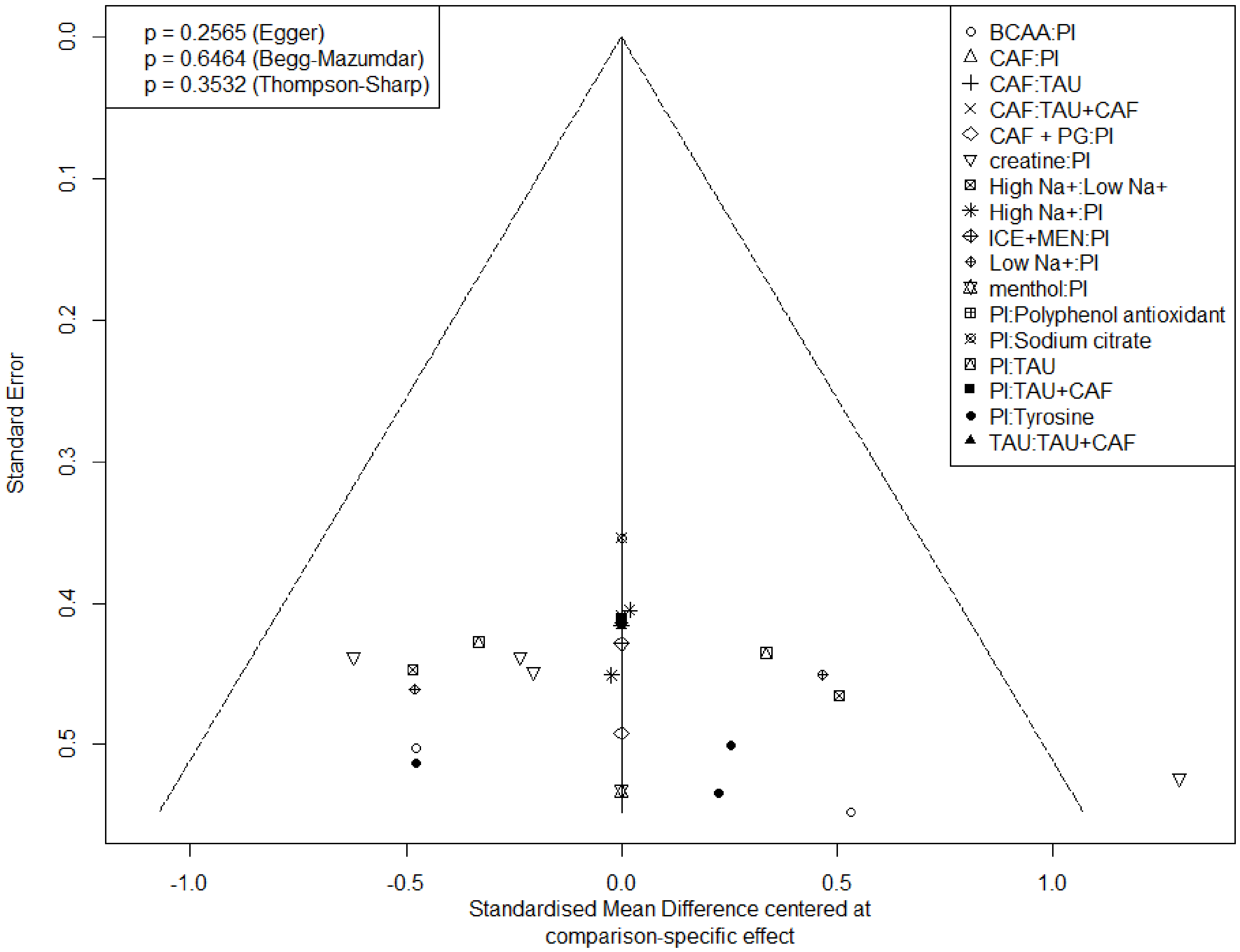

p = 0.2565 (Egger)
p = 0.6464 (Begg-Mazumdar)
p = 0.3532 (Thompson-Sharp)
BCAA:Pl
CAF:Pl
CAF:TAU
CAF:TAU+CAF
CAF + PG:Pl
creatine:Pl
High Na+:Low Na+
High Na+:Pl
ICE+MEN:Pl
Low Na+:Pl
menthol:Pl
Pl:Polyphenol antioxidant
Pl:Sodium citrate
Pl:TAU
Pl:TAU+CAF
Pl:Tyrosine
TAU:TAU+CAF
Standard Error
0.0
0.1
0.2
0.3
0.4
0.5
-1.0
-0.5
0.0
0.5
1.0
Standardised Mean Difference centered at comparison-specific effect

Supplement Figure S4. Iterative and Posterior Distribution Maps of Endurance Performance and Subjective Perception.

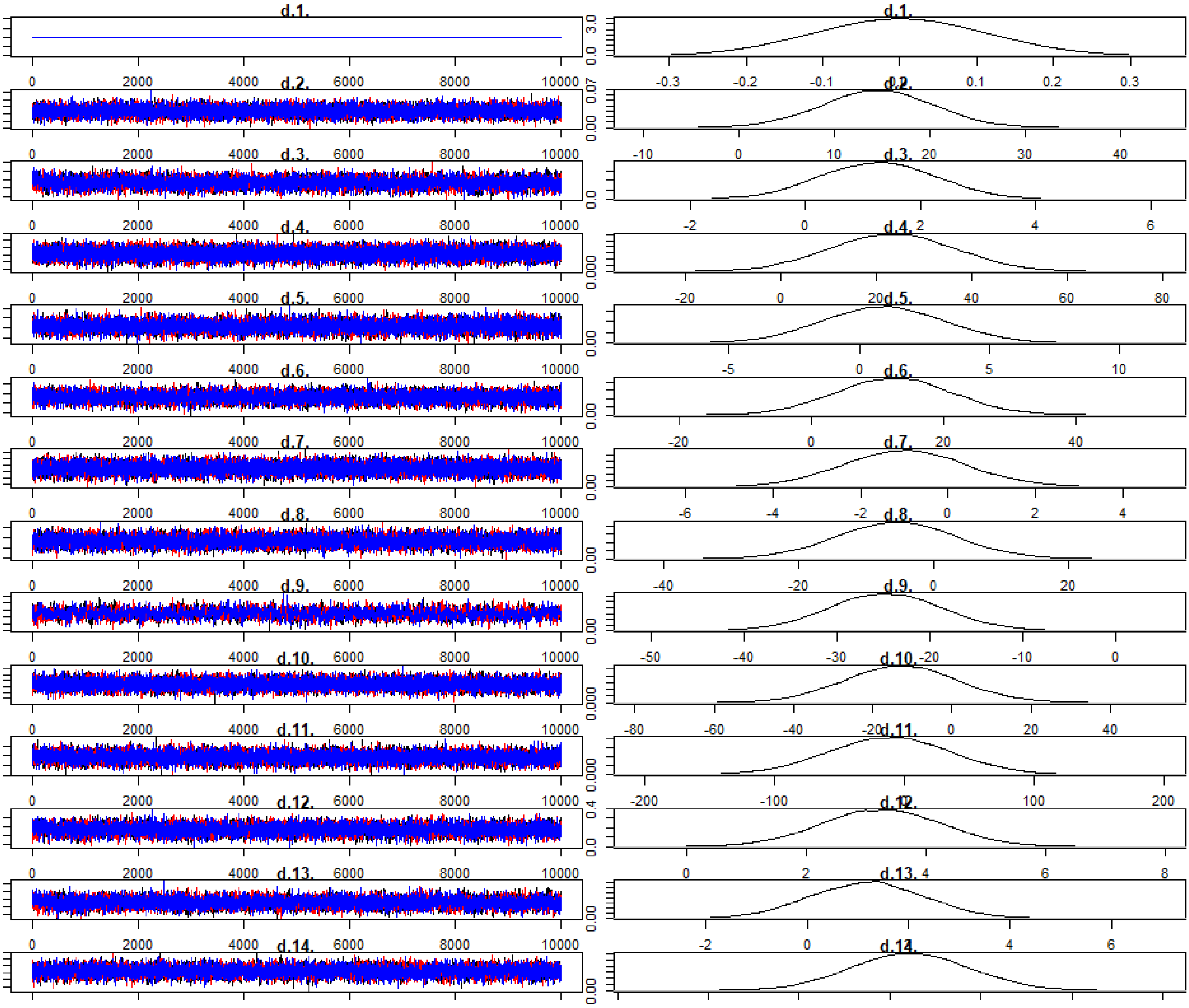

Endurance Performance

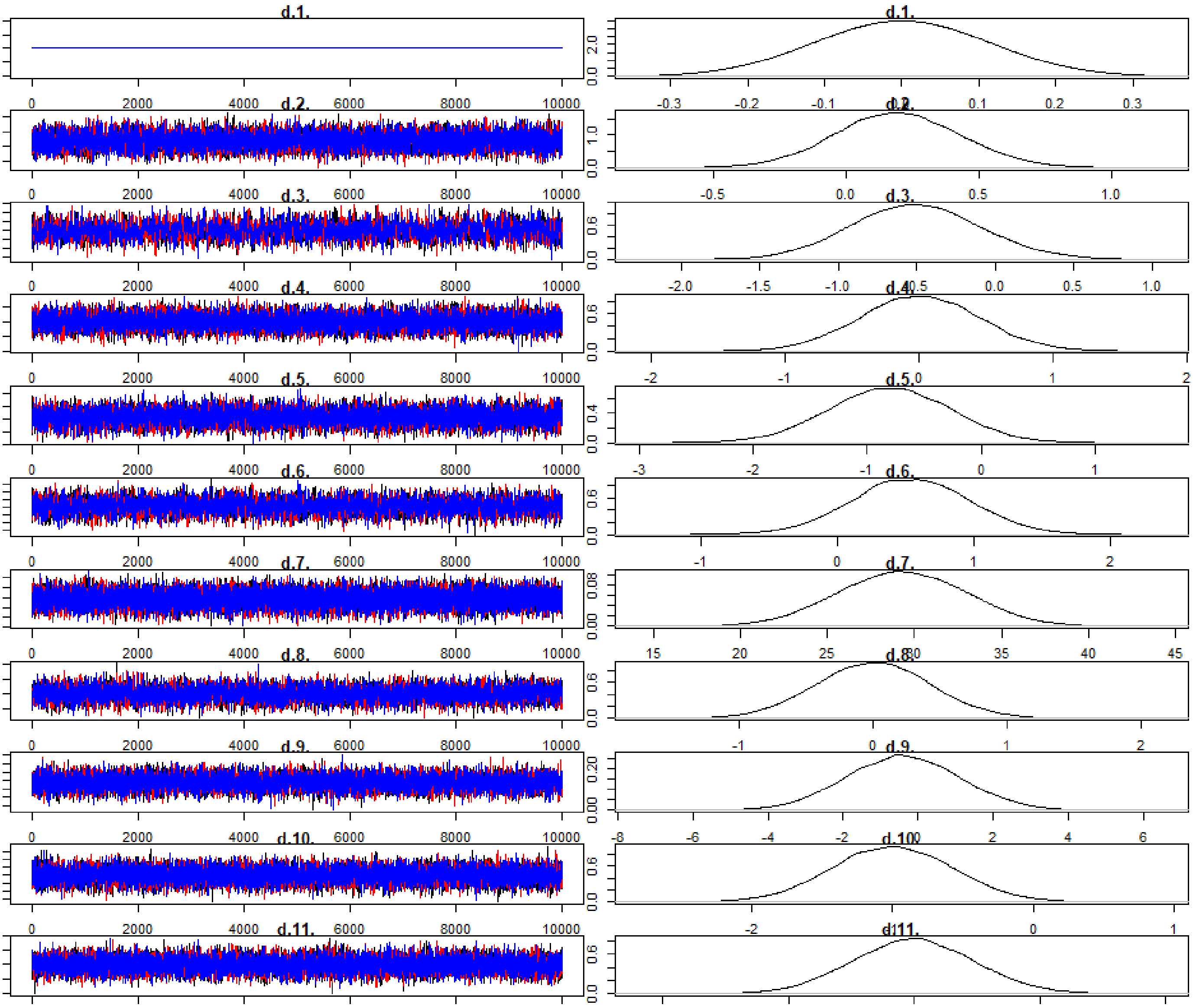

Subjective Perception

Supplement Figure S5. Forest plots of eligible comparisons of Endurance Performance and Subjective Perception.

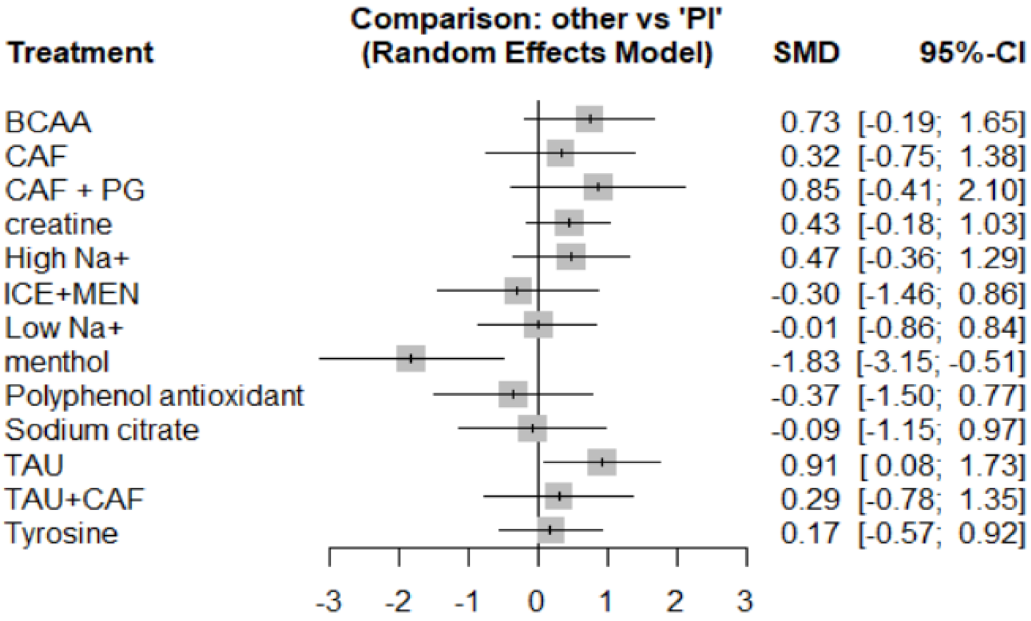

Endurance Performance

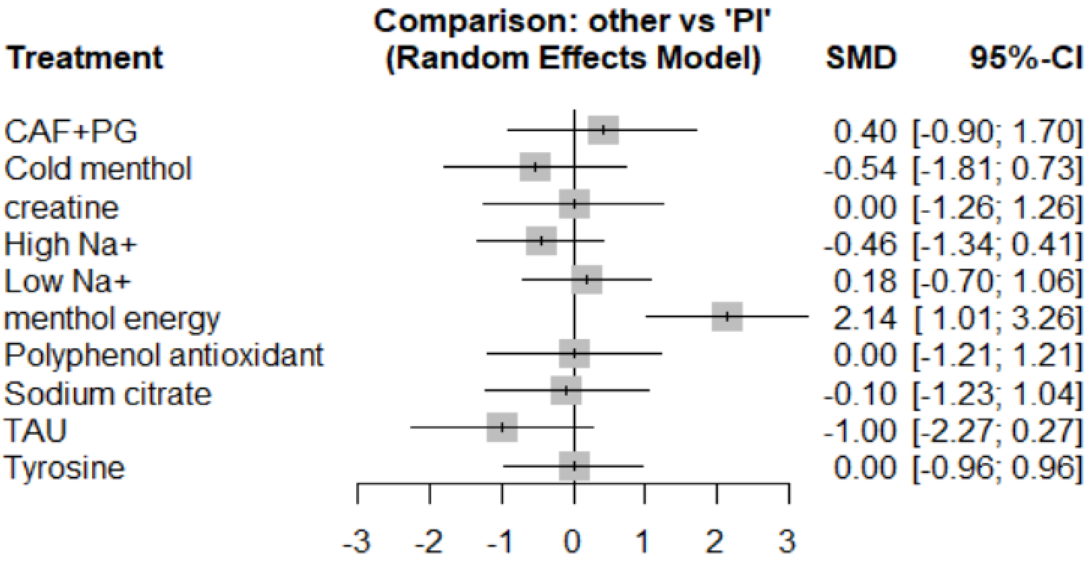

Subjective Perception

Supplement Figure S6. Area under the curve for cumulative ranking probability of each intervention on Endurance Performance and Subjective Perception.

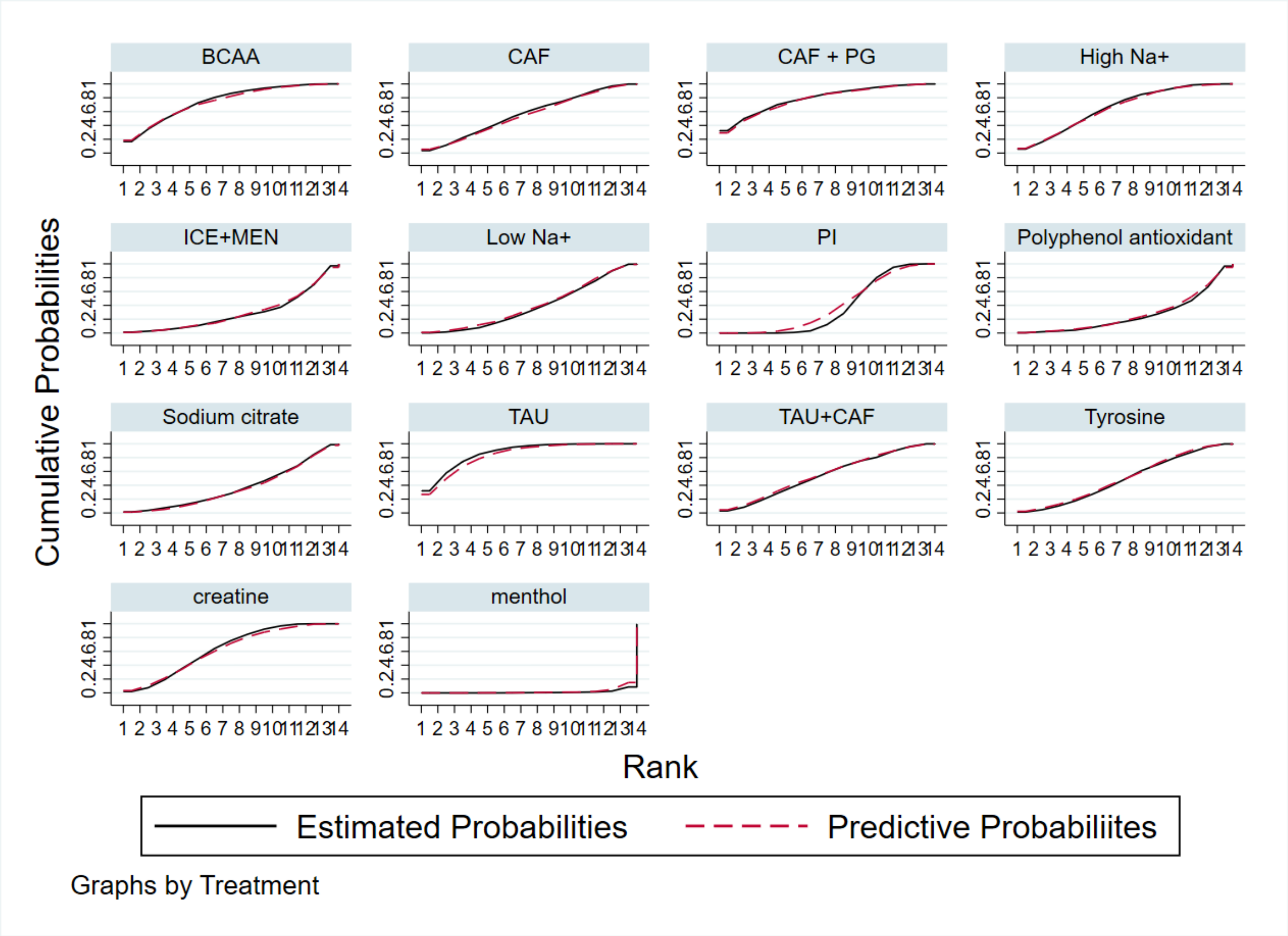

Endurance Performance

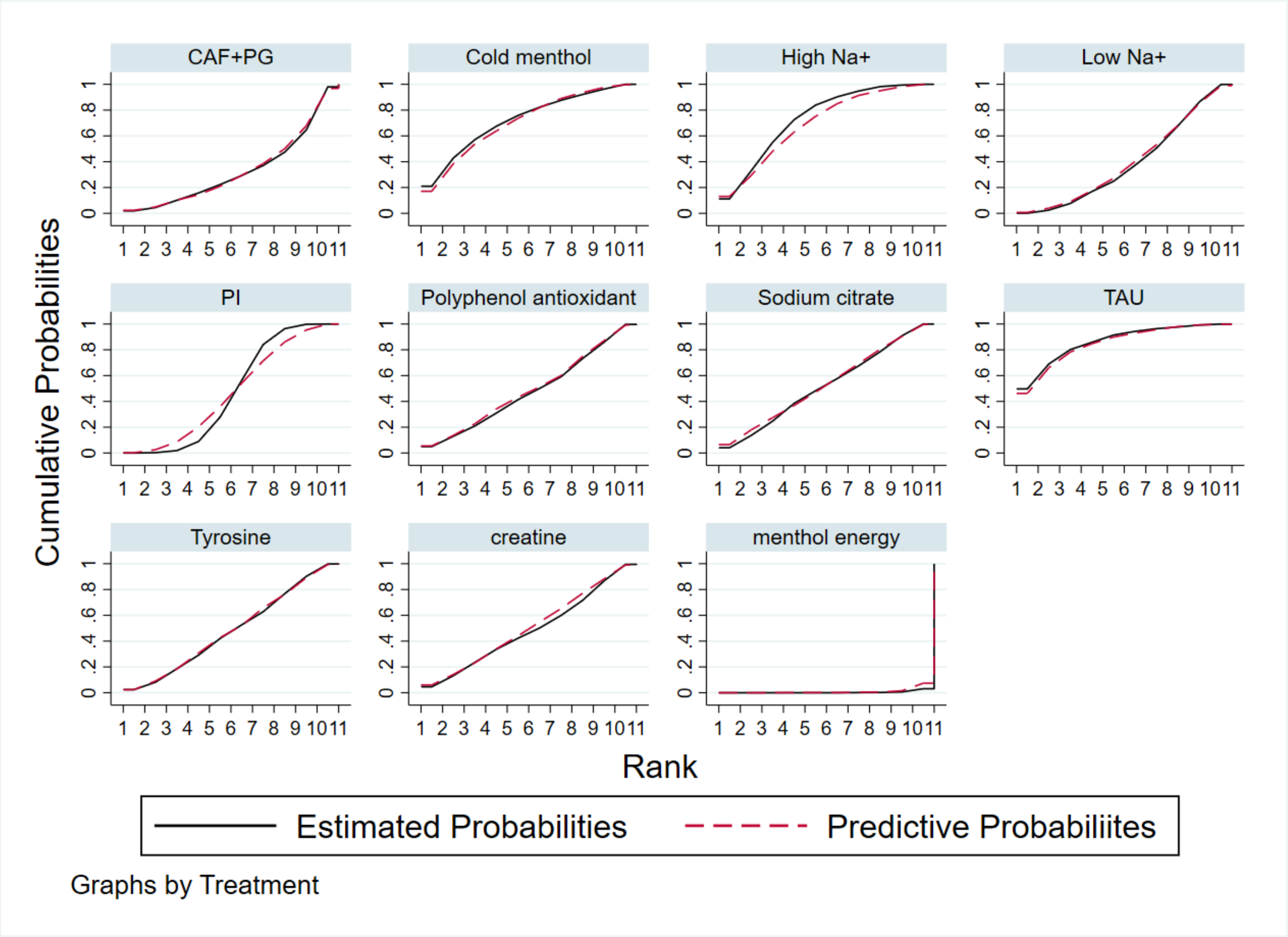

Subjective Perception

Supplement S9. R language source code

```
# Installation Package install.packages("gemtc")

install.packages("rjags")

# Load Package library(gemtc)

library(rjags)

# Clear environment variables and set the path rm(list=ls())

getwd()

setwd("D:/R")

# Data Import mydata<-read.table("mydata2.txt",header=T,na.strings = c("NA"))

# View Data head(mydata,5)

# Conduct a network meta-analysis network<- mtc.network(mydata, description="Example")

model <- mtc.model(network, type = "consistency", factor = 2.5, n.chain = 3, likelihood = "normal", link = "identity", linearModel = "fixed") results <-mtc.run(model, sampler ="rjags", n.adapt =10000, n.iter =100000, thin = 1)

forest(results)

# Creating a Trapezoidal Table tbl <- relative.effect.table(results)

print(tbl)

# Draw convergence plot

pdf(file="gelman_plot.pdf") gelman.plot(results)

dev.off()

tiff(file="gelman_plot.tiff") gelman.plot(results)

dev.off()

png(file="gelman_plot.png") gelman.plot(results)

dev.off()

pdf(file="iteration_and_posterior_distribution_plot.pdf") plot(results)

dev.off()

tiff(file="iteration_and_posterior_distribution_plot.tiff") plot(results)

dev.off()

png(file="iteration_and_posterior_distribution_plot.png") plot(results)

dev.off()

# Node Analysis Draw Node Analysis Forest Chart result.ns <- mtc.nodesplit(network, thin=50,likelihood="normal",link="identity")

summary.ns <- summary(result.ns)

print(summary.ns)

pdf(file="node_analysis.pdf") plot(summary.ns)
```

dev.off()

Sorting

ranks <- rank.probability(results, preferredDirection = -1) # The larger the value, the better. preferredDirection = 1 (for ascending order), preferredDirection = -1 (for descending order) print(ranks)

pdf(file="Rank order diagram.pdf plot(ranks)

dev.off()

Reference

1. Kilduff LP, Georgiades E, James N, Minnion RH, Mitchell M, Kingsmore D, et al. The effects of creatine supplementation on cardiovascular, metabolic, and thermoregulatory responses during exercise in the heat in endurance-trained humans. Int J Sport Nutr Exerc Metab. 2004;14(4):443-60.
2. Wright GA, Grandjean PW, Pascoe DD. The effects of creatine loading on thermoregulation and intermittent sprint exercise performance in a hot humid environment. J Strength Cond Res. 2007;21(3):655-60.
3. Hadjicharalambous M, Kilduff LP, Pitsiladis YP. Brain serotonin and dopamine modulators, perceptual responses and endurance performance during exercise in the heat following creatine supplementation. J Int Soc Sports Nutr. 2008;5:14.
4. Volek JS, Mazzetti SA, Farquhar WB, Barnes BR, Gómez AL, Kraemer WJ. Physiological responses to short-term exercise in the heat after creatine loading. Med Sci Sports Exerc. 2001;33(7):1101-8.
5. Tumilty L, Davison G, Beckmann M, Thatcher R. Oral tyrosine supplementation improves exercise capacity in the heat. Eur J Appl Physiol. 2011;111(12):2941-50.
6. Tumilty L, Davison G, Beckmann M, Thatcher R. Failure of oral tyrosine supplementation to improve exercise performance in the heat. Med Sci Sports Exerc. 2014;46(7):1417-25.
7. Watson P, Enever S, Page A, Stockwell J, Maughan RJ. Tyrosine supplementation does not influence the capacity to perform prolonged exercise in a warm environment. Int J Sport Nutr Exerc Metab. 2012;22(5):363-73.
8. Tran Trong T, Riera F, Rinaldi K, Briki W, Hue O. Ingestion of a cold temperature/menthol beverage increases outdoor exercise performance in a hot, humid environment. PLoS One. 2015;10(4):e0123815.
9. Stevens CJ, Thoseby B, Sculley DV, Callister R, Taylor L, Dascombe BJ. Running performance and thermal sensation in the heat are improved with menthol mouth rinse but not ice slurry ingestion. Scand J Med Sci Sports. 2016;26(10):1209-16.
10. Vogel RM, Ross ML, Swann C, Rothwell JE, Stevens CJ. Athlete perceptions of flavored, menthol-enhanced energy gels ingested prior to endurance exercise in the heat. J Int Soc Sports Nutr. 2022;19(1):580-92.
11. Hamouti N, Fernández-Elías VE, Ortega JF, Mora-Rodriguez R. Ingestion of sodium plus water improves cardiovascular function and performance during dehydrating cycling in the heat. Scand J Med Sci Sports. 2014;24(3):507-18.
12. Sims ST, Rehrer NJ, Bell ML, Cotter JD. Preexercise sodium loading aids fluid balance and endurance for women exercising in the heat. J Appl Physiol (1985). 2007;103(2):534-41.
13. Sims ST, van Vliet L, Cotter JD, Rehrer NJ. Sodium loading aids fluid balance and reduces physiological strain of trained men exercising in the heat. Med Sci Sports Exerc. 2007;39(1):123-30.
14. Yu P, Fan Y, Wu H. Effects of Caffeine-Taurine Co-Ingestion on Endurance Cycling Performance in High Temperature and Humidity Environments. Sports Health. 2024;16(5):711-21.
15. Bandyopadhyay A, Ping FW, Keong CC. Effects of acute supplementation of caffeine and Panax ginseng on endurance running performance in a hot and humid environment. J Hum Ergol (Tokyo). 2011;40(1-2):63-72.
16. Ping FW, Keong CC, Bandyopadhyay A. Effects of acute supplementation of Panax ginseng on endurance running in a hot & humid environment. Indian J Med Res. 2011;133(1):96-102.
17. Watson P, Shirreffs SM, Maughan RJ. The effect of acute branched-chain amino acid supplementation on prolonged exercise capacity in a warm environment. Eur J Appl Physiol. 2004;93(3):306-14.
18. Mittleman KD, Ricci MR, Bailey SP. Branched-chain amino acids prolong exercise during heat stress in men and women. Med Sci Sports Exerc. 1998;30(1):83-91.

19. Cathcart AJ, Murgatroyd SR, McNab A, Whyte LJ, Easton C. Combined carbohydrate-protein supplementation improves competitive endurance exercise performance in the heat. Eur J Appl Physiol. 2011;111(9):2051-61.
20. Easton C, Turner S, Pitsiladis YP. Creatine and glycerol hyperhydration in trained subjects before exercise in the heat. Int J Sport Nutr Exerc Metab. 2007;17(1):70-91.
21. Page LK, Jeffries O, Waldron M. Acute taurine supplementation enhances thermoregulation and endurance cycling performance in the heat. Eur J Sport Sci. 2019;19(8):1101-9.
22. Trinity JD, Pahnke MD, Trombold JR, Coyle EF. Impact of polyphenol antioxidants on cycling performance and cardiovascular function. Nutrients. 2014;6(3):1273-92.
23. Vaher I, Timpmann S, Aedma M, Ööpik V. Impact of acute sodium citrate ingestion on endurance running performance in a warm environment. Eur J Appl Physiol. 2015;115(4):813-23.
24. Fleischmann C, Horowitz M, Yanovich R, Raz H, Heled Y. Asthaxanthin Improves Aerobic Exercise Recovery Without Affecting Heat Tolerance in Humans. Front Sports Act Living. 2019;1:17.
25. Kajiki M, Katagiri A, Matsutake R, Lai YF, Hashimoto H, Nishiyasu T, Fujii N. Ingesting carbonated water post-exercise in the heat transiently ameliorates hypotension and enhances mood state. Exp Physiol. 2024;109(10):1683-97.
